# Supplementary material for: Endothelial dysfunction in ME/CFS patients
Source: PLoS One. 2023 Feb 2;18(2):e0280942. doi: 10.1371/journal.pone.0280942 (PMC9894436; doi:10.1371/journal.pone.0280942)
Supplement: S1 File — (PDF) [file pone.0280942.s001.pdf]

B-LYMPHOCYTE DEPLETION USING THE ANTI-CD20 ANTIBODY RITUXIMAB (MABTHERA®) IN MYALGIC ENCEPHALOMYELITIS/CHRONIC FATIGUE SYNDROME ("RituxME")

A multicentre, randomized, double-blind and placebo controlled phase III study with Rituximab induction and maintenance treatment

Protocol code: KTS-6-2014
EudraCT: 2014-000795-25
ClinicalTrials.gov: NCT02229942

Version	Date	Approved	Description of alterations
1.0	03.03.2014		N/A
1.1	06.05.2014	IEC: 05.06.14	Changes in patient information for main study and the substudy Endothelial function. Details on biobank. Stated name for data manager. New analysis, patient information and control group in the substudy Endothelial function. Adjustments to randomisation process. New personnel and change of treatment location, OUS. Data exchange between Notodden Hospital and HUS. Specification of placebo. Updated procedure for infusion, appendix C. New appendix J5, patient instruction on self-reporting of symptoms.
1.2	02.07.2014	NOMA: 31.07.14	Changes following assessment by the Independent Ethics Committee and the Norwegian Medicines Agency: Specified stop criteria. Changed label for infusion bags. Updated app. C: Procedure for administration of study drug. Specified procedures for preparing, labelling and dispensing study drug, including study drug accountability records for Notodden hospital. Updated study site personnel records.
2.0	22.12.2014	IEC: 19.03.15 NOMA informed 20.03.15	Updated with minor changes to project plan, contact details, collection of routine and biobank blood samples and new clinical monitors. Specified waiting time before inclusion after discontinuation of ME/CFS specific medication. Corrected examinations used for GI substudy. New appendices C, I and K due to minor adjustments.

Signatures (location, date, name)

______________________ ______________________

______________________ ______________________

______________________ ______________________

______________________ ______________________

Table of contents

TRIAL SITES AND RESPONSIBLE STAFF MEMBERS 5
Study management 5
Haukeland University Hospital (HUS) 5
Oslo University Hospital (OUS) 5
Notodden Hospital 5
St. Olav's Hospital 6
The University Hospital of North Norway (UNN) 6
National study coordinator 6
Safety board 7
Local study coordinators 7
Doctors employed in part time positions 7
Medical statistics 8
Immunological analyses 8
Biobank 8
Study monitoring 8
Sensewear armband for activity registration 9
Rituximab/Placebo (saline solution) intervention 9
Staff in charge of sub-studies included in the main clinical study 9
Endothelial function by Flow-Mediated Dilation (FMD) in ME/CFS 9
Ergospirometry in ME/CFS 10
Irritable Bowel Syndrome and functional dyspepsia in ME/CFS 10

BACKGROUND AND PROJECT DESCRIPTION 10
Published pilot study 11
Published double-blind, randomized and placebo controlled study 12
Ongoing open phase study with Rituximab in ME/CFS 13
Planned new randomized phase III study 15
Hypothesis 15

NEW PHASE III STUDY 18
Project plan, project management, organisation and collaboration 19

THE MAIN STUDY OBJECTIVE 20

ENDPOINTS 20
Primary endpoint 20
Secondary endpoints 20

DESIGN 22

PATIENT SAMPLE, POWER ANALYSIS 22

INCLUSION CRITERIA 23

EXCLUSION CRITERIA 23

USE OF OTHER MEDICATIONS 24

PRE-SCREENING AND ASSESSMENT 24

CONSULTATION 25

RANDOMISATION 25

INTERVENTION 25

DATA COLLECTION, DATA MANAGEMENT, STATISTICAL ANALYSIS 26

Modified DePaul and HADS questionnaires *27*
Self-reported symptom score *28*
Self-reported symptom score before intervention *28*
Self-reported symptom change during follow-up *28*
"Total function level" *29*
Self-reported perception of group allocation at 6 weeks *29*
Self-reported overall assessment of development during 24 months follow-up *29*
SF-36 questionnaire on health, and analysis *30*
Fatigue Severity Scale *30*
Doctor's registration at baseline and follow-up, including toxicity *31*
Sensewear armbands for recording activity level at home *31*

MONITORING 32

INFUSION, RITUXIMAB OR PLACEBO 32

EXAMINATIONS AND REGISTRATION IN THE STUDY 34
EXAMINATIONS AND REGISTRATION AFTER SIGNED INFORMED CONSENT, BEFORE INTERVENTION (BASELINE) *34*
Clinical assessment: 34
Laboratory tests 34
Immunology 34
Endocrinology 35
Microbiology 35
Biobank blood tests 35
Sensewear armband for activity registration for 7 consecutive days 35
BASELINE EXAMINATIONS FOR PATIENTS PARTICIPATING IN SUBSTUDIES *35*
Substudy: Endothelial function in ME/CFS 35
Substudy: Ergospirometry in ME/CFS 35
Substudy: Irritable Bowel Syndrome and functional dyspepsia in ME/CFS 36
EXAMINATIONS AND REGISTRATION AT 3, 6, 9, 15, 18 AND 21 MND FOLLOW-UP *36*
Clinical assessment with registration and entry in medical records 36
Laboratory tests 37
Immunology 37
Blood samples for biobank 37
BETWEEN 17 AND 21 MONTHS (ALL PATIENTS) *37*
Sensewear armbands for activity registration for 7 consecutive days 37
BETWEEN 17 AND 21 MONTHS, FOR SUBSTUDY PATIENTS *37*
EXAMINATIONS AND REGISTRATION AT 12 AND 24 MONTHS FOLLOW-UP *37*
Clinical assessment with registration and entry in medical records 37
Laboratory tests 38
Immunology 38
Blood samples for biobank 38
Final visit at 24 months *38*

BIOBANK FOR BIOLOGICAL STUDIES AND IMMUNOPHENOTYPING 38

PATIENT WITHDRAWAL DURING STUDY 39

ADVERSE EVENTS, SAFETY BOARD, SIDE EFFECTS 40
Safety Board and side effects *40*

ETHICAL ASPECTS 42

FUNDING 44

PUBLICATION 44

APPLICATIONS FOR APPROVAL **44**

SUBSTUDIES OF THE MAIN CLINICAL STUDY **45**
SUBSTUDY: ENDOTHELIAL FUNCTION IN ME/CFS *45*
Flow-mediated Dilation (FMD) 45
Microvascular endothelial function 46
SUBSTUDY: ERGOSPIROMETRY IN ME/CFS *47*
SUBSTUDY: IRRITABLE BOWEL SYNDROME AND FUNCTIONAL DYSPEPSIA IN ME/CFS *49*

REFERANSER **50**

APPENDICES

A. Canadian criteria for ME/CFS.
B. Modified DePaul questionnaire for prescreening before inclusion.
C. Directions for rituximab (Mabthera®)/placebo infusions.
D. Self-report form for ME/CFS symptoms before intervention.
E. Self-report form for ME/CFS symptom change every second week.
F1. SF-36 (v1.2) health questionnaire.
F2. Fatigue Severity Scale (FSS) questionnaire.
F3. Hospital Anxiety and Depression Scale (HADS) questionnaire.
G. Form for doctor’s registration of ME/CFS at baseline and follow-up.
H. Serious Adverse Events and CIOMS report forms.
I. Blood samples for biobank and immunophenotyping.
J1. Patient information and consent form for main clinical study
J2. Patient information and consent form for substudy: Endothelial function
J2.1. Patient information and consent form for substudy: Endothelial function, control group
J3. Patient information and consent form for substudy: Ergospirometry
J4. Patient information and consent form for substudy: IBS/functional dyspepsia
J5. Patient instructions on self-reporting
K. Flow charts for clinical study and substudies

TRIAL SITES AND RESPONSIBLE STAFF MEMBERS

Study management

The study is managed from the Dept. of Oncology and Medical Physics at Haukeland University Hospital (HUS), by Head of Dept., Professor Olav Mella and Senior Consultant Øystein Fluge.

Coordinating investigator for the clinical study

Olav Mella, MD, PhD, Professor, Head of Dept.
Dept. of Oncology and Medical Physics,
Haukeland University Hospital, 5021 Bergen
Tel: +47 55972069
Mobile: +47 90990185
E-mail: olav.mella@helse-bergen.no
E-mail: olav_mella@hotmail.com

Haukeland University Hospital (HUS)

PI, Øystein Fluge, MD, PhD, Senior Consultant
Dept. of Oncology and Medical Physics,
Haukeland University Hospital, 5021 Bergen
Tel.: +47 55972010
Mobile: +47 93044024
E-mail: oystein.fluge@helse-bergen.no
E-mail: oystein.fluge@gmail.com

Oslo University Hospital (OUS)

Patient screening, inclusion, intervention and follow-up will take place at the Dept. of Medicine, OUS Ullevål, in collaboration with the ME/CFS centre, Dept. of Medicine, OUS Aker.

PI (OUS) Katarina Lien, MD
Oslo University Hospital HF
Aker Hospital
The ME/CFS Centre
Postboks 4959 Nydalen
0424 Oslo
Tel: +47 22851558
Mobile: +47 91807034
Email: katarina.lien@medisin.uio.no
Email: katarinalien@gmail.com

Notodden Hospital

PI Hanne Thürmer, MD, PhD, Senior Consultant
Dept. of Medicine, Notodden hospital
Henrik Wergelandsgate 9, 3675 Notodden
Tel.: +47 35021109
Mobile: +47 90151761

E-mail: hanne.thurmer@live.no

St. Olav's Hospital

PI Petter Chr. Borchgrevink, MD, PhD, Professor
Dept. of Pain and Complex Disorders
St. Olav's Hospital
Postboks 3250 Sluppen
7006 Trondheim
Tel.: +47 72822500
Mobile: +47 91198840
E-mail: petter.borchgrevink@ntnu.no

The University Hospital of North Norway (UNN)

PI Christoph Schäfer, MD, PhD, Chief Senior Consultant
Division of Rehabilitation Services
University Hospital of Northern Norway
Pb 1, 9038 Tromsø
Tel.: +47 77626316
Mobile: +47 46509664
E-mail: christoph.schaefer@unn.no

National study coordinator

Kari Sørland, BA, RN
Dept. of Oncology and Medical Physics,
Haukeland University Hospital
5021 Bergen
Tel: +47 55970439
Mobile: +47 47719398
E-mail: kari.sorland@helse-bergen.no
E-mail: karisorland@hotmail.com

In collaboration with:

The Clinical Trial Unit, Haukeland University Hospital
Mari Holsen, study nurse and Marianne Lehmann, study nurse
Tel.: +47 55972890
E-mail: mahh@helse-bergen.no
marianne.emblem.lehmann@helse-bergen.no

NorCRIN
Project Coordinator Ingvill Finnes, Pharmacist
St. Olav's Hospital
Postboks 3250 Sluppen
7005 Trondheim
Tel.: +47 72820615
Mobile: +47 91182442
E-mail: ingvill.finnes@st.olav.no

Safety board

Olav Dahl, Professor, Senior Consultant, Dept. of Oncology and Medical Physics, Haukeland University Hospital (Chairman)
Ola Didrik Saugstad, Professor, Senior Consultant, Institute of Paediatrics, University of Oslo
Unn Merete Fagerli, PhD, Senior Consultant, Dept. of Oncology, St. Olav's Hospital

Tel. (Olav Dahl): +47 55972018
Mobile: +47 91884617
E-mail: olav.dahl@helse-bergen.no

Local study coordinators

Dept. of Oncology and Medical Physics,
Haukeland University Hospital:
Study nurse Kari Sørland
Tel.: +47 55970439
Mobile: +47 47719398
E-mail: kari.sorland@helse-bergen.no

Dept. of Medicine, Notodden hospital:
Study nurse Ann Elin Lonar
Tel.: +47 35021124
Mobile: +47 97189366
E-mail: ann.elin.lonar@sthf.no

The CFS/ME centre, Oslo University Hospital:
Study nurse Sissel Skulberg Martinsen
Tel.: +47 23031861
E-mail: smartins@ous-hf.no

Dept. of Pain and Complex Disorders, St. Olav's Hospital:
Study nurse Ann Elise Havnen Solvang
Tel.: +47 72822547
Mobile: +47 90010542
E-mail: ann-elise.havnen.solvang@stolav.no

Division of Rehabilitation Services, UNN:
Study nurse Arne Edvard Sørli Gya
E-mail: arne.edvard.sorli.gya@unn.no

Doctors employed in part time positions

Haukeland University Hospital: Ingrid Gurvin Rekeland
Oslo University Hospital: Ingrid Herder
Notodden Hospital: Mohpal Singh Kahlon
St. Olav's Hospital: Katarzyna Baranowska
The University Hospital of North Norway: Louis Bohnen

Medical statistics

Analyses of study data will be executed in collaboration with Section for Medical Statistics, University of Bergen.

Randomisation will be performed by medical statistician Nils Smeland at Smerud Medical Research International AS.

Immunological analyses

Head of Dept. Einar K. Kristoffersen, Professor
Dept. of Transfusion Medicine and Immunology
Haukeland University Hospital, 5021 Bergen
Tel.: +47 55974683
E-mail: einar.kleboe.kristoffersen@helse-bergen.no

Biobank

Ove Bruland, PhD
Centre for Medical Genetics and Molecular Medicine
Haukeland University Hospital
5021 Bergen
Tel.: +47 55975324
E-mail: ove.bruland@helse-bergen.no

Kine Alme, M.Sc.
Dept. of Oncology and Medical Physics,
Haukeland University Hospital
5021 Bergen
Tel.: +47 55976255
E-mail: kine.alme@helse-bergen.no

Sigrid Lunde, M.Sc.
Dept. of Oncology and Medical Physics,
Haukeland University Hospital
5021 Bergen
Tel.: +47 55976255
E-mail: sigrid-lunde@helse-bergen.no

Kristin Risa, Cand. Scient
Dept. of Oncology and Medical Physics,
Haukeland University Hospital
5021 Bergen
Tel.: +47 55976255
E-mail: kristin.risa@helse-bergen.no

Study monitoring

Clinical monitor for HUS and UNN:
Ingunn H. Anundskås,
Innovest AS
Møllendalsveien 65 C
5009 Bergen

Tel.: +47 55970955
E-mail: ingunn.heie.anundskaas@helse-bergen.no

Clinical monitor for OUS and Notodden:
Nina Flatner
Dept. for clinical research support
OUS Ullevål
Tel.: +47 22119054
E-mail: ninafl@ous-hf.no

Clinical monitor for St. Olav:
Trude Langeng
Dept. of research
St. Olav's Hospital
Tel.: +47 91186029
E-mail: trude.langeng@stolav.no

Sensewear armband for activity registration

Armbands will be sent to all participants from the Oncology Dept., HUS. Sensewear armbands are returned in a pre-paid envelope and will be analysed at HUS, by Espen K. Krohn-Hansen and Tor Helge Wiestad in collaboration with study nurse Anne Falch.

Address: Anne Falch
Dept. of Oncology and Medical Physics,
Haukeland University Hospital
5021 Bergen
Tel.: +47 55972069
E-mail: anne.falch@helse-bergen.no

Rituximab/Placebo (saline solution) intervention

At 0 and 2 weeks (induction), and at 3, 6, 9 and 12 months (maintenance); IV infusions of rituximab/ saline solution ***(appendix C)***.

Infusions will be administered:

- At HUS: the Oncology Dept. outpatient clinic (Helle M. Øvrebø, Oncology Nurse).
- At Notodden hospital: the Chemotherapy outpatient clinic (Dr. Erling Dahl Borkamo).
- At the Dept. of Medicine outpatient clinic, OUS Ullevål.
- At St. Olav's Hospital: the Oncology Dept. outpatient clinic (Dr. Øivind Kvammen).
- At UNN: Division of Rehabilitation Services.

Staff in charge of sub-studies included in the main clinical study

Endothelial function by Flow-Mediated Dilation (FMD) in ME/CFS

This sub-study will be executed at Haukeland University Hospital and at Notodden hospital. Patients'' FMD is measured at baseline, and after 17-21 months ***(please refer to sub-study section of protocol)***.

- At HUS: Miriam K. Sandvik, MD, PhD, and Elisabeth Leirgul, MD, PhD, Senior Consultant in Cardiology.

E-mail: miriamsandvik@gmail.com
elisabeth.leirgul@gmail.com

- At Notodden Hospital: Hanne Thürmer, MD, PhD, Senior Consultant in Cardiology.
E-mail: hanne.thurmer@live.no

-At Haukeland University Hospital microvascular function will also be measured using Laser-Doppler method (Periflux-5000), at baseline and at 17-21 months ***(please refer to sub-study section of protocol)***.
Analyses will be performed by study nurse Kari Sørland and medical student Christina Stavland.

Ergospirometry in ME/CFS

Execution of this sub-study is planned at OUS, HUS and Notodden Hospital.
For patients with mild/moderate illness who can bear the strain of such exercise, at baseline and at 17-21 months follow-up ***(please refer to sub-study section of protocol)***.

-At OUS: Katarina Lien, MD
E-mail: katarina.lien@medisin.uio.no
katarinalien@gmail.com

-At HUS: Exercise therapists Tor Helge Wiestad and Espen K. Krohn-Hansen.
E-mail: tor.helge.wiestad@helse-bergen.no

-At Notodden hospital: Hanne Thürmer, MD, PhD, Senior Consultant in Cardiology.
E-mail: hanne.thurmer@live.no

Irritable Bowel Syndrome and functional dyspepsia in ME/CFS

Execution of this sub-study is planned for patients included at HUS.
Patients included in the main clinical study who experience gastrointestinal symptoms can be invited to participate. Patients are examined at baseline and at 17-21 months follow-up ***(refer to sub-study section of protocol)***.

Managed by Prof. Trygve Hausken, Prof. Odd Helge Gilja and Dr. Elisabeth K. Steinsvik
Dept. of Medicine, Section of Gastroenterology
Haukeland University Hospital
E-mail: trygve.hausken@helse-bergen.no
E-mail: odd.helge.gilja@helse-bergen.no

BACKGROUND AND PROJECT DESCRIPTION

Myalgic Encephalomyelitis/Chronic Fatigue Syndrome (ME/CFS) is characterised by pathological exhaustion and malaise, particularly after strenuous activity, in conjugation with cognitive symptoms such as difficulties with concentration and

memory, sensory hypersensitivity, pain (typically muscle and joint pain and new-onset headaches), sleep disturbances as well as a variety of symptoms from the autonomous nervous system. ME/CFS affects approximately 0.1-0.2 % using strict diagnostic criteria [1], and must be differentiated from more general fatigue, which affects a larger percentage of the population. Patients with serious ME/CFS have considerably reduced quality of life, and the condition carries great public socio-economic costs. In recent years, several findings have been published supporting immune dysregulation [2,3], abnormalities in the composition of cerebrospinal fluid [4], reduced cerebral circulation [5] and changes in EEG [6]. Of particular relevance to our study, an epidemiological study has shown that elderly ME/CFS patients are at an increased risk of developing B-cell-derived non-Hodgkin lymphoma [7].This susceptibility to B-cell-derived lymphoma has also been observed in other conditions where chronic immune activation is the recognized pathological process. The cause of ME/CFS is unknown, and no universally accepted and effective treatment exists. A lack of reliable biomarkers means the diagnosis is mainly based on the patient's own experience of the illness. Diagnostic criteria are used to separate the ME/CFS patients from other conditions characterized by general fatigue [8].

At the Oncology Dept. at Haukeland University Hospital a patient observation was recorded. The patient was diagnosed with Hodgkin's lymphoma, but also had a 7 year long history of stable and debilitating ME/CFS. She experienced a recurrence of her HL after her primary treatment, and after several chemotherapy regimens including high-dose treatment with autologous stem cell transplant, she has now been recurrence-free for 7 years. During one of the chemotherapy treatment regimens (MIME), she experienced a significant improvement in all ME/CFS related symptoms, which started 6-7 weeks after commencing chemotherapy. Her ME/CFS symptoms gradually relapsed after 5 months of remission. She reported/experienced no change in ME/CFS symptoms during the other chemotherapy regimens that she received. The MIME regimen, to which her ME/CFS symptoms appear to respond, contains a relatively low dose of methotrexate plus ifosfamide. We speculated that a relative B-cell depletion, such as can be seen after low-dose weekly methotrexate treatment of e.g. rheumatoid arthritis, might thus have been the cause of the improvement in ME/CFS symptoms.

As the current knowledge of the use of B-cell depletion to target symptom maintenance in ME/CFS is limited to our two complete and two ongoing studies, we will describe these studies in more detail.

Published pilot study

The above-mentioned patient and another two pilot patients with ME/CFS were treated with the monoclonal anti-CD20 antibody rituximab (Mabthera®), which depletes B-lymphocytes effectively and selectively. All three pilot patients had significant but limited duration responses affecting their entire ME/CFS symptomatology [9]. The first two pilot patients had an "early response pattern", showing improvement from approx. 6-7 weeks after infusion, with response duration of 3-4 months. The third patient had a "late response pattern", showing significant improvement from approx. 22 weeks after infusion and a similar response duration of 4 months followed by gradual relapse. Subsequent experience has shown that the "late" response pattern is the most common pattern.

Published double-blind, randomized and placebo controlled study

The Oncology Dept. at Haukeland University Hospital has, in collaboration with the Dept. of Neurology, conducted a double-blind and placebo controlled study with 30 patients. Specifically, half of the patients were given two infusions of rituximab 500mg/ m^2 with two weeks' interval, and the other half were given the equivalent two infusions with saline solution. Patients were followed for 12 months (KTS-1-2008). The study was published in PLoS One [10]. Inclusion was based on the Fukuda diagnostic criteria [11]. 70 % of the patients were women, the average age was 37 years in the rituximab group and 32 years in the placebo group, and average duration of symptoms was 5 years in the rituximab group and 8 years in the placebo group. For 70 % of the patients, the ME/CFS onset was preceded by an evident or probable infection. The prevalence of co-existing autoimmune conditions in the patients was 23 %, and among first-degree relatives was 40%, which is higher than would be expected in the general population.

Whilst performing this study a link between a retrovirus Xenotropic murine leukaemia virus-related virus (XMRV) and ME/CFS was published [REF]. We therefore assessed all participants and found none had detectable XMRV. Several subsequent studies have refuted the connection and have also been unable to detect XMRV in ME/CFS patients resulting in the original Science publication being retracted. [10].

Monitoring of response was achieved by patients recording a report of their symptoms every two weeks during the follow-up period (starting after the first infusion), compared to their own baseline. The report contained symptoms connected with pathological exhaustion (fatigue), cognitive function, pain, and "other symptoms" including sleep disturbances, gastrointestinal symptoms, hypersensitivity to sound and light, abnormal perspiration, palpitations and a sore throat. A symptom score was calculated every second week, based on the patient's report. Short Form 36 (SF-36 v1.2), which is a standard validated questionnaire for health-related quality of life, was completed every month.
Overall response was defined from the self-reported Fatigue score. Statistical analysis for repeated measurements of Fatigue score showed a significant interaction between time after treatment and intervention group (p=0.018). In other words, the Fatigue score development was significantly different in favour of the rituximab group, indicating the treatment was improving fatigue whilst the placebo wasn't. The differences between the groups were most evident from 6-10 months after intervention, correlating with the secondary endpoint. During protocol design the assumption of maximum response at 3 months was based on the experience with the first two pilot patients with «early» responses, while the most common pattern we subsequently observed was the «late» response. Hence the difference between the groups was most pronounced at 8 months after intervention. As a result, the primary end point, which was predefined as 3 months after intervention, was negative.

Overall response, defined (exploratively and post-hoc) as significant and lasting improvement in Fatigue score was registered in 10 patients in the rituximab group

(67%, 95% CI 41%-85%) and in only 2 patients in the placebo group (13%, 95% CI 4%-38%) (p=0.003). The average response duration within the 12 months follow-up period for the 10 responders in the rituximab group was 25 weeks (duration 8-44). Four patients had response durations beyond the study period (12 months), and after more than 4 years two patients are still in complete remission. One patient in the placebo group is also registered as a lasting responder.

There were no serious infections or other serious side-effects, but two patients with pre-existing psoriasis experienced a moderate worsening in their psoriasis symptoms which coincided in both cases with an improvement in ME/CFS-related symptoms. 1/3 of the rituximab group had no response after B-cell depletion with two infusions of rituximab with two weeks' interval. We proposed that for some non-responders the duration of the B-cell depletion period may have been too short. In the next study, two of the non-responders from this study were included. A total of 29 patients were given two infusions with two weeks' interval, followed by maintenance infusions after 3, 6, 10 and 15 months. One of the two non-responders that participated in the subsequent trial with the different treatment regime did experience a significant response of all ME/CFS related symptoms. This suggests that for some patients, response occurs in a dose-dependent manner. For some patients, however, there were no sign of response after 12 months follow-up. These patients were classified as non-responders and may represent a sub-group within the ME/CFS population.

Ongoing open phase study with Rituximab in ME/CFS

The open phase II study (KTS-2-1010, no placebo group) was brought to a conclusion in February 2014. The study investigated the dose-response relationship of Rituximab in ME/CFS treatment. By February 2011, a total of 27 patients had been included (as well as two patients from the previous study – see above). Rituximab was administered as two IV infusions with two weeks' interval (corresponding to the randomized study), followed by maintenance infusions of Rituximab after 3, 6, 10 and 15 months. Following protocol amendment approved by the Research Ethics Committee, a total of six patients who after 12 months follow-up were experiencing a clinical response, but a slow and gradual improvement in ME symptoms, have received up to 5 additional Rituximab infusions, resulting in protracted B-cell depletion. The following preliminary results are quoted in confidence. All patients have now completed a minimum of 36 months follow-up, and approx. 70 % have experienced clinical improvement (response) according to pre-defined criteria. A major response was detected in 14 patients, a moderate response in four patients, and a "marginal" response in three patients. These responses were defined as major, moderate or marginal based on the self-reported symptoms recorded fortnightly during follow-up, changes in the quality of life questionnaire SF-36, the patients' own perception and the doctors' clinical assessment. The patients with "marginal" response met predefined response criteria, but the response duration was short and occurred late in the follow-up period, and is presumed to be unrelated to the intervention. Seven patients (25 %) experienced no response.

In the 14 patients with major response, we have seen a considerable change in the pattern of symptoms, usually involving an improvement in all ME/CFS-related symptoms, and with an average response duration of 108 weeks (during the study period of 36 months). In the four patients with moderate response, the average

response duration was 68 weeks. At study conclusion (36 months), 12 out of 21 responders showed continued response, whilst the remaining 9 have experienced various degrees of relapse during the last year. One pilot patient is still in complete response 54 months after inclusion in the study. It seems clear that maintenance treatment with repeated infusions of Rituximab cause a significantly increased response duration compared to the observed response duration after two Rituximab infusions alone[10]. While the published double-blind study can be considered a "proof of principle", the ongoing study with Rituximab induction and maintenance provides information on the extent of continued major response that might be achieved through B-cell depletion.

In addition to the fortnightly, self-reported symptom scores and the SF-36 quality of life questionnaire, the patients also recorded their experienced total function level, expressed as a percentage (where 100 % correlates to their healthy state, i.e. the patient's condition before ME/CFS onset), before intervention (baseline) during the best four week period between 10 and 15 months and between 18 and 24 months follow-up, and finally at study conclusion (36 months). For the 21 responders, the average baseline value (before intervention) was 14% (on a scale of 0-100) compared to an average value for best period between 10 and 15 months of 65%, between 18 and 24 months of 74%, and between 30 and 36 months of 53%. The equivalent values for the 7 non-responders were an average 19% before intervention, and 23% for the best 4 week periods between 10 and 15 months and also for 18 and 24 months.
The 21 patients with response based on pre-defined criteria recorded an average SF-36 raw score (average for sub-dimensions Physical Function, Bodily Pain, Vitality, General Health and Social Function, scale 0-100) of 30 at baseline, 57 after 15 months, 65 after 24 months, and 58 after 36 months. The differences in SF-36 scores from baseline to the three time points are highly significant (adjusted for several comparisons).
The results clearly indicate that most patients who experience response after Rituximab treatment achieve a significant improvement in their function level. Nevertheless, per February 2014 approx. one third of the patients are either non-responders or have recorded marginal "response" after Rituximab maintenance treatment. In addition, approx. half of the major and moderate responders suffer various degrees of relapse after 36 months follow-up.

With regards to side effects, one patient suffered an allergic reaction (non-anaphylactic) to Rituximab during the first infusion, and consequently did not receive further infusions. After maintenance treatment with a total of 6 Rituximab infusions and a minimum of 36 months follow-up, two patients experienced an episode of late-onset neutropenia, both of which were uncomplicated and with a duration of 5 days. This is a common complication which affects approx. 5 to 10% of lymphoma patients treated with Rituximab [12]. From between one to two years' follow-up, two patients had recurrent respiratory infections requiring several courses of antibiotic treatment. Both were given normal human immunoglobulin (Kiovig®) with effect on the tendency toward infections. One patient has had an upper urinary tract infection resulting in hospitalisation for one day. Adverse Events not clearly related to the Rituximab intervention were recorded. One patient had a gallstone attack with jaundice after 33 months follow-up, and was treated with endoscopic papillotomy.

One patient discovered a lump in the breast after 24 months, and was diagnosed with breast cancer (T2N0M0, ER+) and treated with surgery (ablation) and postoperative adjuvant chemotherapy and endocrine therapy. One patient, who was a non-responder in the trial, was diagnosed with idiopathic thrombocytopenic purpura (ITP), an autoimmune disease, shortly after the end of follow-up at 36 months (27 months after last Rituximab infusion).

We are planning to publish data from this open phase II study during 2014.
An open phase II study with Rituximab induction and maintenance treatment as described above is also being conducted with up to 15 patients with very severe ME/CFS (KTS-3-2010). By February 2014, eight patients were included in this study, four of which have been almost constantly bedridden for years. We have experienced great logistical challenges transporting these very ill patients to a hospital, and find it difficult in a busy oncology ward to provide the level of seclusion required. Moderate response is recorded in one of the eight patients, while two (very severely ill) have experienced a beneficial effect on the symptoms without satisfying the response criteria. Our impression based on these dates is that patients with very severe illness are less likely to respond to B-cell depletion using Rituximab. For this reason severely affected patients will not be included in a new randomized phase III study.

Planned new randomized phase III study

The published study in Plos One [10] has limitations. It was explorative in nature, being the first study to examine B-cell depletion as a principle of treatment in ME/CFS. The endpoint for expected response in the protocol turned out to be too early and the study was small. In order to confirm or disprove the association of B-cell depletion with statically and clinically significant responses in ME/CFS the study must therefore be repeated in larger patient groups, with a more optimal Rituximab dosage interval and predefined end points based on our more recent experience.

The Research Council of Norway has now granted financial support for the execution of a new randomized, double-blind, placebo controlled, multi-centre national study. This study will compare treatment with Rituximab or saline solution; two infusions with two weeks' interval (500 mg/m2, max. 1000 mg), followed by maintenance infusions of Rituximab/saline solution after 3, 6, 9, 12 months (500 mg, fixed dosage). The double-blind design will be maintained until the last included patient has been assessed at 24 months follow-up.

Hypothesis

Our hypothesis is that ME/CFS is caused by a form of immune system dysregulation, often triggered after infections. The mechanism could be a type of autoimmune or autoinflammatory process. This assumption is based on the course of response and relapse of ME/CFS symptoms following B-cell depletion. While the B-cells are reduced to very low levels in peripheral blood within days or weeks after commencing Rituximab infusions, there is a «delay» of 2 to 11 months before initial clinical responses are reported. We propose that this delayed response pattern could correlate with a gradual elimination of (auto)antibodies.

In the recently completed phase II study with Rituximab maintenance treatment, the average time from the first Rituximab infusion until start of clinical response was 22

weeks (minimum 8 weeks, maximum 50 weeks) for the 14 patients with major responses. The equivalent figure for four patients with moderate response was an average 55 weeks (from 28 to 64 weeks before start of clinical response).
The response rates as well as the course of response and relapse are consistent with observations following Rituximab treatment in auto-immune conditions such as rheumatoid arthritis. The overrepresentation of women, a proven genetic predisposition [13], and the occurrence of other autoimmune illnesses in the family of ME/CFS patients are other factors that all suggest a possible immunological pathogenesis. A proven increased risk of B-cell lymphoma in elderly ME/CFS patients [7] also suggests that the patients have a chronically activated B-cell system. This is an evolving hypothesis, and there is ongoing laboratory work being carried out in order to investigate the aetiology and pathogenesis of this disease.
B-cell depletion is an intervention that causes significant disruption to the coordinated action of the immune system, and several other possible interpretations to our findings could therefore be relevant. The effect could be related to reinforcement of Th1-responses after Rituximab treatment and reduction of active B-cells, or due to non-B cell mediated effects such as an impact upon T-cell antigen presentation, or on the regulation of other effector cells in the innate immune system such as monocytes/macrophages or dendritic cells [14]. It is also possible that the effect is related to the elimination of B-lymphotrope viruses such as the Epstein Barr virus (EBV) caused by B-cell depletion.

B-cell depletion in ME/CFS patients has resulted in varying responses, from no response (1/3), via moderate response, to major response. Some patients experience significant changes, elimination of all symptoms and a sense of full recovery. Generally, we have observed that all ME/CFS related symptoms are affected during response, including fatigue related symptoms, cognitive symptoms, pain and ”other symptoms”. This indicates that treatment with Rituximab affecting a central pathogenesis either directly or indirectly.
The available data indicate that B-lymphocytes are essential to symptom maintenance in a subgroup of ME/CFS patients. In these patients the temporal course of response and relapse can be viewed as compatible with a fundamental immunodysregulation, where the elimination of immunoglobulins contributes to symptom alleviation. The determination of a target for this type of immune-mediated process will be crucial to the understanding of the ME/CFS pathogenesis. Furthermore the identification of the target could be used as a starting point for the identification of a specific biomarker, and may open up novel therapeutic targets for effective symptomatic treatment, which interferes directly with the effector system for symptom maintenance.

Over time, we have reached an understanding that the considerable subjective symptoms suffered by ME/CFS patients, from various organ systems, is probably associated with the dysfunction of an extensive biological system. A dysfunction in such an extensive effector system could generate the wide range of symptoms presented by these patients.
A study measuring reactive vasodilation of arteria brachialis after 4-5 min. occlusion using a blood pressure cuff (flow mediated vasodilation, FMD), concludes that ME/CFS-patients have endothelial dysfunction [15].

In collaboration with the Department of Cardiology at HUS, we have measured endothelial function using FMD in a total of 16 ME/CFS patients. Average FMD was 3.5%, and five patients had an FMD < 1%. This is in comparison to an average FMD of 8.5% measured in healthy women, using the same equipment and protocol, by the same two doctors. Only one out of 66 healthy women had an FMD < 2%. Thus our preliminary data support the findings in the above-mentioned study [15].
Endothelial dysfunction is a risk factor for cardiovascular diseases [16], and a slight to moderate reduction in FMD is also associated with autoimmune systemic diseases [17]. A slight or moderate association between FMD and depression is also described in some studies [18].
Thus the preliminary data from our analyses in ME/CFS patients show a significantly reduced FMD, which could be an essential discovery with implications for the pattern of symptoms found in this disease. A main attribute of endothelial dysfunction measured with FMD is an inadequate nitrogen monoxide (NO) synthesis in endothelial cells.
Based on knowledge of several functions of NO we propose that a relative lack of NO could contribute to the ME/CFS symptoms. NO causes vasodilatation, and is an important factor in the autoregulation of blood flow, where “shear stress” in the vessel wall constitutes an important signal for eNOS activation. The enzyme eNOS is a catalyst for the production of NO in endothelial cells. Amongst the many functions of NO that appear to be of relevance to ME/CFS symptoms NO is a neural transmitter that can affect memory and concentration. In addition, NO relaxes smooth muscle cells and changes the motility in the gastrointestinal tract and the urogenital system, and NO affects platelet aggregation and the contractility of the heart [19]. NO also affects the immune system, where iNOS regulates the activity of macrophages, T-lymphocytes, antigen-presenting cells, mast cells, neutrophil granulocytes and NK cells [20]. Low levels of NO cause sensory hyperexcitability, particularly affecting Kv channels (voltage-gated potassium channels), in which an increase in NO provides control over neuronal excitability [21,22]. ”Spillover” from the various sources of nitrite (endothelial cell eNOS, neuron nNOS, immune cell iNOS contribute to the total level of NO, in a complex interplay, which is very hard to regulate with drug interventions. It is likely that nitrate/nitrite measured in plasma as an expression for NO metabolites (NOx) reflects the iNOS-derived NO [23]. While the low, tonic, constitutive NO synthesis from endothelial cells (eNOS) and neuron cells (nNOS) stays in the nanomolar region, inducible iNOS will increase the NO-production by 1000 to micromolar region.

A number of data from the literature relating to ME/CFS can be explained by a hypothesis that involves a dysregulated NO system as a contributory effector system for symptom maintenance. Several studies have shown increased lactate levels in cerebrospinal fluid in patients with ME/CFS[24,25]. Furthermore, a local increase in lactate levels has been detected in cerebral tissue of ME/CFS patients after mental and physical strain, using MR spectroscopy amongst other techniques. Repeated stress tests on two consecutive days show that ME/CFS patients reach anaerobic threshold at a lower level of exercise and importantly at a low oxygen uptake. The patients have lower scores on day 2, which are also associated with an increased lactate production from anaerobic glycolysis and reduced mitochondrial ATP production [26]. A recently published study shows that reduced oxygen extraction from blood in

ME/CFS patients and a concomitant reduction in oxygen uptake in muscle cells and probably other tissue as well [27]. Reduced NK-cell function is shown in several studies on ME/CFS patients [28].

Regulation of blood flow in vivo is very complex and involves an array of mediators and the coordinated action of the autonomous nervous system [29]. However, Flow-Mediated Dilation (FMD) executed under standardised conditions adequately reflects the endothelium's ability to produce NO when exposed to increased shear stress from the blood flow [30].
We hypothesise that the symptom maintenance in a subgroup of ME/CFS patients is partly caused by a relative lack of NO bioavailability from endothelial cells. If this is the case, the inadequate regulation of blood flow in response to tissue oxygenation and nutrition requirements will have a fundamental effect on the pattern of symptoms. The next step must be to elucidate what connection there is between the effect of B cell depletion and the effector system with endothelial dysfunction and relative endothelium derived NO unavailability.
The clinical responses after rituximab treatment, starting at the earliest 2 months after the first infusion, suggests the possible involvement of an antibody (long half-life), and the immune response interfering with a signalling pathway which eventually adds up to a disturbed endothelial function, indirectly or directly. If this hypothesis is correct, i.e. if ME/CFS is a variant of an autoimmune disease where an auto-antibody is involved, the existence of a target with an important normal function in regulating endothelial and eNOS activity and possibly also other functions such as uptake of glucose and oxygenation of tissue, might explain the pathogenesis of the disease. Regulation of the eNOS-complex is a complex process, with several positive and negative regulators are known [31].

In 1/3 of the patients treated with rituximab there is no clinically significant response. It is possible that other mechanisms independent of the B-lymphocytes can also cause endothelial dysfunction and a similar clinical presentation.
Uncovering the aetiology and pathogenesis of ME/CFS is imperative. There needs to be a specific test or biomarker that could identify the group of patients with probable immunological affliction. These will be the patients who can be expected to profit from pharmaceutical intervention targeting the immune system. As the first trial [10] was small and had exploratory elements with several weaknesses, and the next trial with rituximab induction and maintenance treatment is an open study without a placebo group, there is a need for a more robust trial. A randomised, double-blind and placebo controlled, multi-centre study needs to be performed in order to verify or disprove the association of B-lymphocyte depletion using the monoclonal anti-CD20 antibody rituximab with clinically significant responses in a larger cohort of ME/CFS patients. Verification of such an association could lead to the approval of treatment of ME/CFS patients with rituximab, and thus hope of relief and improved function and quality of life for a large group of patients currently suffering.

NEW PHASE III STUDY

B-LYMPHOCYTE DEPLETION USING THE ANTI-CD20 ANTIBODY RITUXIMAB (MABTHERA®) IN MYALGIC ENCEPHALOMYELITIS/CHRONIC FATIGUE SYNDROME ("RituxME")

A multicentre, randomized, double-blind and placebo controlled phase III study with Rituximab induction and maintenance treatment

Project plan, project management, organisation and collaboration

Following approval from the Regional Ethics Committee and the Norwegian Medicine's Agency, the study commenced in the third quarter of 2014.
The randomisation code will be broken after the last included patient has completed a 24 month follow-up period. If the inclusion phase lasts for 9 months, the randomisation code can be broken 33 months after study start date.
Assuming that the results show that the intervention with rituximab is associated with clinically significant responses in ME/CFS patients, those patients allocated to the placebo group will, after the randomisation code has been broken, be offered participation in a new study involving rituximab. This will also be dependent on the availability of funding.

The study has a multicentre design. The main trial site will be at the Dept. of Oncology and Medical Physics at Haukeland University Hospital (HUS), managed by Coordinating Investigator, Head of Dept. Prof. Olav Mella, and Project Manager, Senior Consultant Øystein Fluge, MD.
Patients will be included at Oslo University Hospital (OUS) Ullevål by Katarina Lien, MD, at the University Hospital of North Norway (UNN) by Senior Consultant Christoph Schaefer, at St. Olav's Hospital by Prof. Petter Chr. Borchgrevink and Senior Consultant Merethe Eide Gotaas, and at Notodden Hospital by Senior Consultant Hanne Thürmer.

The Dept. of Clinical Research at HUS will, in collaboration with the Coordinating Investigator Olav Mella, PI Øystein Fluge and National Study Coordinator Kari Sørland, be responsible for follow-up of the trial sites, ensure Good Clinical Practice (GCP) compliance, and be responsible for data collection.

NorCRIN (the Norwegian Clinical Research Infrastructure Network) will contribute during project planning and conduction. NorCRIN will create the Trial Master File and Investigator Study Files, draft agreements with suppliers, take part in project meetings and produce newsletters – all in collaboration with the study management.

The study will be carried out in accordance with the Norwegian regulations: "Regulation relating to clinical trials on medicinal products for human use" (FOR 2009-30-10) and Good Clinical Practice (GCP) guidelines.

Data analyses will be performed in collaboration with the Section for Medical Statistics at the University of Bergen. The study will be monitored externally by Ingunn H. Anundskås at Innovest AS.

THE MAIN STUDY OBJECTIVE

The objective of the study is to verify or disprove the association of B-cell depletion using the monoclonal anti-CD20 antibody rituximab (Mabthera®) with clinically significant responses in ME/CFS patients.

ENDPOINTS

Primary endpoint

The primary endpoint is recorded from the patient self-report form (***appendix E***) completed every two weeks during a follow-up period of minimum 24 months. The variable Fatigue score is calculated every two weeks as the mean (scale 0-6) of the four symptoms: Fatigue, Post-exertional malaise, Need for rest and Daily function. The mean Fatigue score for each patient during the specific time intervals in the follow-up period (0-4, 4-8, 8-12, 12-16, 16-20, 20-24 months), will be subject to statistical analysis (General Linear Model (GLM) for repeated measures), where the difference in Fatigue score over time (24 months) by intervention group (rituximab group and placebo group), forms the primary endpoint [10].

The Overall Response records the effect on the ME/CFS symptoms during 24 months after intervention start date. The Overall Response is not predefined to a specific time interval during the 24 months of follow-up, but the response must be recorded as moderate or major on the patient self-report form. Overall Response is defined as mean Fatigue score ≥ 4.5 for a minimum of 8 consecutive weeks for moderate response, and including a mean Fatigue score ≥ 5.0 for a minimum of 8 consecutive weeks for major response. The duration and sum of the various response periods during the 24 months will be recorded.

Secondary endpoints

-SF-36 scores (”Physical health summary score”,”Mental health summary score” and scores for eight SF-36 subdimensions) are analysed at baseline and at 3, 6, 9, 12, 15, 18, 21 and 24 months ***(appendix F1)***.
Changes in the SF-36 ”Physical health summary score” (norm based), the SF-36-subdimension ”Physical Function” (raw score) and changes in mean scores for the five SF-36 subdimensions ”Physical Function”, ”Bodily Pain”, ”Vitality”, ”Social Function” and ”General health” (raw scores), from baseline to the predefined time points during follow-up, will be recorded.
Difference in SF-36 ”Physical Health summary score”, SF-36 ”Physical Function” and mean SF-36 scores of five subdimensions over time (24 months follow-up) by intervention group (rituximab group and placebo group), will be analysed using GLM for repeated measures and constitute a secondary endpoint.

Changes will also be recorded for the same SF-36 scores (”Physical health summary score”,”Physical Function” (raw score), and the mean score for five subdimensions (PF, BP, GH, V, SF), from baseline to 12, 15, 18, 21 and 24 months follow-up. Changes from baseline to the individual time points for rituximab and placebo groups can be analysed using Repeated Measures One-way ANOVA (adjusted for multiple comparisons).

The difference between the rituximab and placebo groups for changes in ”Physical health summary score”, ”Physical Function” raw score, and the mean score for five subdimensions (PF, BP, GH, V, SF), from baseline to 18 months follow-up will constitute a secondary endpoint.

-Changes in physical activity measured by a Sensewear armband for seven consecutive days, before intervention and again between 17 and 21 months after intervention start date. Changes will be recorded for mean number of steps per 24 hours, max. Number of steps per 24 hours, mean duration of moderate activity ≥ 3.5 METs per 24 hours, max. Duration of moderate activity ≥ 3.5 METs per 24 hours.
The difference between the rituximab and placebo groups in changes from baseline to 17-21 months after intervention start date, for mean number of steps per 24 hours and mean duration of moderate activity ≥ 3.5 METs per 24 hours, will constitute a secondary endpoint.

-”Total function level” (scale 0-100, compared to healthy state) is recorded in the patient’s self-report form ***(appendix E)*** every two weeks. The mean ”Total function level” is recorded for time intervals 0-4 months, 4-8 months, 8-12 months, 12-16 months, 16-20 months and 20-24 months follow-up, and will serve as input for GLM for repeated measures, which can be used to compare the self-reported “Total function level” over time (24 months follow-up) by intervention group. This constitutes a secondary endpoint.

-Changes from baseline in self-reported Fatigue score over the last 4 months and changes from baseline in self-reported ”Total function level” over the last 4 months, at 8, 12, 16, 20 and 24 months after intervention start date ***(appendix E)*** will be recorded.
The difference between rituximab and placebo groups in changes in Fatigue score and Total function level score from baseline to the time interval 16-20 (mean) constitutes a secondary endpoint.

-The Fatigue Severity Scale (FSS) will be completed at baseline, and at 6, 12, 18 and 24 months. Changes in FSS score from baseline and throughout follow-up will be recorded (***appendix F2***).
The difference between rituximab and placebo groups in changes in FSS score from baseline to 18 months follow-up constitutes a secondary endpoint.

-The longest duration of lasting clinical response defined as lasting self-reported Fatigue score ≥ 4,5 (at least 8 consecutive weeks) during the 24 month follow-up period is recorded. Differences between the rituximab and placebo groups will be analysed.

-The number of patients who have recorded response according to the response criteria and who show no sign of relapse (lasting Fatigue score ≥ 4.5 at 24 months follow-up), will be registered. Differences between the rituximab and placebo groups will be analysed.

DESIGN

Multicentre, national, randomised, double-blind and placebo-controlled phase III study.

PATIENT SAMPLE, POWER ANALYSIS

A total of 152 patients with chronic fatigue syndrome (ME/CFS) will be included. The patients must be between 18 and 66 years old, with disease duration of between 2 and 15 years. For patients with a mild degree of ME/CFS, disease duration must be at least 5 years.
Patients must have an established ME/CFS diagnosis according to ”Canadian criteria” [8] ***(appendix A).***

We are planning the inclusion of 40 patients at HUS, 32 patients at OUS Ullevål, 32 patients at Notodden hospital, 24 patients at St. Olav’s Hospital and 24 patients at UNN.
Patients may suffer from ”Mild”, ”Mild/Moderate”, ”Moderate”, ”Moderate/Severe” or ”Severe” ME/CFS. The ME/CFS symptoms and loss of function in the individual patient must be severe enough to justify therapeutic B-lymphocyte depletion with rituximab induction and maintenance treatment, from both the patient’s and doctor’s perspective.
Patients with “Very severe” ME/CFS (completely bedridden and in need of care, WHO class IV) will not be included in the study. Symptom severity will be recorded on the doctor’s registration form before inclusion in the study (***appendix G***). In addition, the trial participants must not have been exposed to rituximab previously.

In the published study in Plos One [10], 2 out of 15 patients (13%) in the placebo group reported a clinical response during the 12 month follow-up period. Due to the small number of patients, there is a broad 95% confidence interval for 13% response in the placebo group (4% - 38%).

Sample power analyses (for Overall Response) show that if 79 patients are included in each group, with an expected “response” rate of 25% in the placebo group and 50% in the rituximab group, and a 5% drop-out rate (8 patients, missing data), the study will have a 90% power to detect a significant difference (alpha 0.05), provided that there is a difference between the groups.
At corresponding response rates and 85% power, we will need 68 patients in each group. With 152 included patients (76 in each group) the statistical power to detect a significant difference will be 85-90%, with a 5% drop out rate.

Self-report form for symptom change during follow-up, from which the Fatigue score is calculated and analysed as the primary endpoint, is designed with a response variable of seven ordinal categories where 3 means unchanged from baseline, 4-5-6 mean slight, moderate and major improvement respectively and 2-1-0 mean slight, moderate and major worsening respectively.

Estimated distribution of symptom change in rituximab and placebo groups:

Response	*0 Major worsening*	*1 Moderate worsening*	*2 Slight worsening*	*3 Unchanged*	*4 Slight improvement*	*5 Moderate improvement*	*6 Major improvement*
**Rituximab**	3%	4%	7%	21%	15%	15%	35%
**Placebo**	5%	5%	7%	46%	12%	15%	10%

Assuming a number of 76 included patients in each group, an estimated 8 drop outs (5% missing data) and the above distribution of the response categories, the study will have a statistical power of 90%, which is the statistical probability that the study will show a statistically significant difference provided that there is a real difference between the groups. With this distribution of response categories, moderate or major improvement will be achieved by 50% in the rituximab group and 25% in the placebo group. Based on our two prior clinical studies, where only the first was placebo-controlled, the estimates for response rates in the rituximab and control groups are uncertain, with wide confidence intervals.

The patients who are invited for evaluation for inclusion in the study will mainly be recruited from available lists of patients with diagnosed ME/CFS at HUS, OUS or the other trial sites, including patients who have contacted the sites, who are interested in participating in the study and who satisfy the inclusion criteria.

INCLUSION CRITERIA

-Patients with ME/CFS according to Canadian criteria of 2003 [8].
-Disease duration: 2-15 years.
-For patients with mild ME/CFS disease duration must be a minimum of 5 years.
-Severity: Mild, Mild/Moderate, Moderate, Moderate/Severe and Severe ME/CFS.
-Age: 18-65 years.
-Signed informed consent.

EXCLUSION CRITERIA

-Patients with fatigue, who do not comply with the diagnostic (”Canadian”) criteria for ME/CFS or disease duration < 24 months or > 15 years.
-Patients where the workup uncovers other pathology as a possible cause of symptoms.
-Patients with very severe ME/CFS (WHO function class IV), who are totally bedridden and in need of care.
-Pregnancy or breast feeding. Positive pregnancy test.
-Previous cancer (except basal cell carcinoma of the skin or cervix dysplasia).
-Previous long-term systemic treatment with immunosuppressive agents (Imurel, Sandimmun, Cellcept), except steroid treatments for e.g. obstructive lung disease or other autoimmune diseases like ulcerative colitis.
-Serious endogenous (primary) depression.
-Lack of ability to complete the study including follow-up.
-Known serious multi-allergy, clinically assessed with an elevated risk of allergic reactions during rituximab infusion.
-Reduced kidney function (creatinine > 1.5 x reference area).
-Reduced liver function (bilirubin > 1.5 x reference area, or transaminase > 1.5 x reference area).

-Known HIV-positivity, previous hepatitis B or hepatitis C, or reason to suspect other ongoing and clinically relevant infection.
-Known immunodeficiency disorders with an elevated risk involved in therapeutic B-lymphocyte depletion, e.g. hypogammaglobulinemia.

USE OF OTHER MEDICATIONS

Candidates may not be included while undergoing Gammanorm or other immunoglobuline treatment. Immunoglobuline treatment must be discontinued at least three months before inclusion in the study.

If a participant suffers recurring bacterial infections (usually upper respiratory infections) during the study, they may receive therapeutic, intravenous human gammaglobuline such as Kiovig® subject to clinical assessment. This would not result in exclusion for patients who are already included in the study.

Potential candidates who are undergoing treatment with low dose Naltrexone (LDN), GcMAF, Isoprinosine (Immunovir ®) or prolonged antibiotics treatment may be considered for inclusion in the study, but the treatment must be discontinued at least 4 weeks before clinical assessment and pre-inclusion laboratory tests.

Treatment with vitamin B12 which was initiated less than three months before clinical assessment should be discontinued. If the treatment has been ongoing for more than three months, the patient may continue the B12 treatment throughout the study.

If a candidate is using other medications directed at his/her ME/CFS, the study management must be consulted before inclusion.

PRE-SCREENING AND ASSESSMENT

After publication of the PLoS One article in October 2011, we have received more than a thousand letters and e-mails from patients and their families, and around 800 applications for participation in a new study. Patients who seem to fulfil the inclusion criteria, including the Canadian criteria for ME/CFS [8], based on information in medical records or referral letters, can be selected for assessment.
Each trial site will be responsible for inclusion of patients, and the local PI may choose to select candidates from existing patient records, or from newly referred patients.
At HUS there will be an element of random selection between the patients who appear to satisfy the inclusion criteria based on the available information. The selected candidates will then be offered a consultation and medical assessment including a workup as described in the protocol, in order to determine suitability for inclusion.

CONSULTATION

The patients will attend their respective study centres for consultation and assessment, and distribution of a written patient information letter/declaration of consent. No study specific analyses or tests should be performed prior to written informed consent from the patient.
In accordance with the protocol, candidates will be invited for clinical assessment, laboratory and other tests prior to intervention, such as blood samples for basic workup and for the biobank, and Sensewear activity registration for 7 consecutive days.

Patients who are participating in the sub-studies associated with the main clinical study, will attend the relevant examinations (endothelial function – FMD and microcirculation, ergospirometry for two consecutive days and gastrointestinal assessment and tests) ***(refer to separate sub-study section in the protocol)***.
Separate patient information letters and declarations of consent will be distributed for each sub-study.

RANDOMISATION

Statistician Nils Smeland at Smerud Medical Research International AS will perform the randomization of patients. 152 patients will be allocated 1:1 to the rituximab or placebo group by block randomization, with a block size of 8. I.e. for every group of 8 patients included, 4 will be allocated to each group in random order. Randomisation will be performed before study start date at Smerud MRI, and the local hospital pharmacy at each trial site will receive a list of randomised ID numbers. When a new patient is included at the local trial site, this patient will be allocated a study ID-number, and the pharmacy will produce the correct intervention (rituximab or placebo) according to the randomisation list and label the medicine with the study specific label.

All infusions, including induction at 0 and 2 weeks and maintenance after 3, 6, 9 and 12 months, will be administered intravenously ***(appendix C)***.
The randomisation code will be broken after the last included patient has completed 24 months follow-up. After the randomisation code has been broken patients allocated to the placebo group will be offered participation in a new study involving rituximab, if the results show that intervention with rituximab is associated with clinically significant responses in ME/CFS patients, and provided that funding can be obtained.

INTERVENTION

Two intravenous infusions with the monoclonal anti-CD20 antibody rituximab (Mabthera®, 500 mg/m^2, max 1000 mg per infusion), or saline with added Albumin for the placebo group, will be administered with a two week interval as induction treatment. For the two induction infusions we will allow a flexibility of +/- 4 days, i.e. the second infusion is administered 10-18 days after the first.
The maintenance treatment consists of rituximab (Mabthera®, 500 mg fixed dose) (or saline with added Albumin for the placebo group) after 3, 6, 9 and 12 months. For maintenance infusions we will allow a flexibility of +/- 10 days relative to the time

lapsed after intervention start date. Intravenous infusions follow a standardised production set-up at the pharmacy, with study specific labelling of the rituximab/placebo, and all six infusions will be administered over approx. 4 hours (***appendix C)***.

DATA COLLECTION, DATA MANAGEMENT, STATISTICAL ANALYSIS

Data manager is study coordinator Kari Sørland.
When including a patient, the trial sites will contact the study coordinator Kari Sørland (tel. 55970439, mobile 47719398) for central registration. If study coordinator Sørland is not available, the sites will contact the Clinical Research Unit at HUS (tel. 55972890). All included patients will be allocated a study ID number, consisting of a trial site code followed by a patient number (e.g.: 1-04 means patient no. 4 included at trial site 1).

Trial site 1: Oslo University Hospital (Study ID no: 1-01, 1-02, …, 1-32).
Trial site 2: Notodden Hospital (Study ID no: 2-01, 2-02,…, 2-32).
Trial site 3: Haukeland University Hospital (Study ID no: 3-01, 3-02, …, 3-40).
Trial site 4: St. Olav's Hospital (Study ID no: 4-01, 4-02, …, 4-24).
Trial site 5: The University Hospital of North Norway (Study ID no: 5-01, 5-02, ..5-24).

The patients will receive individual study folders. The study folder must be brought to each visit at the trial site. The folder contains (separated by partitions) a front page with contact information and a "checklist/calendar", a copy of the written patient information/consent form, a form for self-reporting of symptoms at baseline (scale 1-10), a form for self-reporting of symptom change (scale 0-6) and total function level (scale 0-100) every two weeks during follow-up, SF-36 quality of life questionnaire to be completed after 0, 3, 6, 9, 12, 15, 18, 21 and 24 months, Fatigue Severity Scale (FSS) questionnaire to be completed after 0, 6, 12, 18 and 24 months, form for self report after 24 months regarding the overall course of symptom development throughout the study.

At every clinical assessment visit, the relevant pages of the self-report form (completed every two weeks since last visit) are photocopied. The original remains in the patient folder. Completed SF-36 life quality questionnaire from the patient folder is collected (no copy in patient folder). At visits after 6, 12, 18 and 24 months Fatigue Severity Scale (FSS) form is also collected (no copy in patient folder).
The collected original forms and copies of self-report form are stored in the patients' individual case file at the trial site.
Data from the forms will be entered into the program Viedoc®, a designated computer system for multi-centre studies which comply with all requirements from the Data Protection Office, the Norwegian Medicines Agency (NOMA), and international agencies such as the US Food and Drug Administration (FDA).

At every site the study coordinator will be responsible for data entry. All trial sites will have access to entered data from their own included patients.

The Clinical Research Unit at HUS will be the responsible site for receipt and verification of data from the trial sites, and national study coordinator Kari Sørland and study nurses at the Clinical Research Unit trained in GCP will perform ongoing quality controls of the data. The study management at HUS (Olav Mella, Øystein Fluge, Kari Sørland and the Clinical Research Unit) will have access to entered data from all patients.

In addition to the full time study coordinator at the Oncology dept., HUS (Kari Sørland), we intend to fund part time positions (50 %) for study coordinators at OUS, St. Olav's Hospital, UNN and Notodden hospital.
If data entry at a trial site is delayed by more than 4 weeks, a reminder will be issued from the study management at HUS.
Paper CRFs are stored at the local trial site. Upon completion of the study, the patient's folder must be handed in at the local trial site and filed according to regulations. Forms with original data will be stored at the trial sites for 15 years after final report is issued. All computer files will be stored accordingly. At each clinical visit, the doctor must dictate an entry in the hospital's electronic medical journal.
The randomisation code will be broken for all patients simultaneously 24 months after intervention start date for the last patient.
SPSS and Graphpad Prism may be used for statistical analysis.

The demographic and clinical characteristics of the patient groups will be described. We will register changes over time for each individual patient, and for groups of patients. Statistically and clinically significant improvement will be assessed.

Data which could reveal group allocation (immunophenotyping of lymphocyte subpopulations in peripheral blood) will be stored at the laboratory (Dept. of Transfusion Medicine and Immunology by Head of Dept. Einar K. Kristoffersen) until the randomisation code has been broken, and will as such remain unavailable to study personnel with patient contact during the follow-up period (until the last included patient has completed 24 months follow-up).

Data from FMD examinations (endothelial function sub-study) performed at Notodden hospital will be de-identified and stored on a CD. This data will be sent by registered mail to the study coordinator at HUS and stored in the study archive. Analyses of FMD data from Notodden and HUS will be performed by Dr. Miriam Sandvik and Dr. Elisabeth Leirgul.

*****

Modified DePaul and HADS questionnaires

Before inclusion all patients will complete a modified DePaul questionnaire ***(appendix B)*** in order to evaluate the patients who, according to the initial information in referral letters or medical journals, appear to satisfy the inclusion criteria including the Canadian criteria for ME/CFS.
The patients also fill in the Hospital Anxiety and Depression Scale (HADS) questionnaire for survey of symptoms related to anxiety and depression (***appendix F3***). The HADS questionnaire consists of 7 questions related to anxiety and 7 questions related to depression, with four alternative answers (scale 0-3) for each

question [32]. HADS is thoroughly validated and frequently used in studies in order to clarify any existing component of anxiety or depression. The patients will only complete the HADS questionnaire at baseline as part of the workup before intervention.

Self-reported symptom score

As there are no definite diagnostic laboratory tests or other specific markers for the disease, patient symptoms are key variables for adequate registration of clinical effect. The effect variables are related to the four main symptom categories in ME/CFS: ”Fatigue”, ”Pain”, ”Cognitive symptoms”, and ”Other symptoms ” (including sleep disturbances, sensory hypersensitivity and symptoms from the autonomous nervous system).

Self-reported symptom score before intervention

Each patient will complete a registration form (after inclusion, before intervention) with scores (scale 1-10) for the patient’s present symptoms, and a score for «Total function level» which is stated as a percentage of a completely healthy state (i.e. 100 %), guided by a set of examples in the patient’s study folder ***(appendix D)***.
Only symptoms which are relevant for the individual patient (i.e. it transpires from the registration form that these symptoms are actually affecting this particular patient) will be analysed for changes during follow-up.

Self-reported symptom change during follow-up

Each patient will fill in the self-report form for symptom change every two weeks, until 24 months follow-up is complete. Symptom change is compared to status before intervention (baseline) throughout the follow-up period.

Changes in symptom severity (scale 0-6, where 3: unchanged, 4: slight improvement, 5: moderate improvement, 6: major improvement, 2: slight worsening, 1: moderate worsening, 0: major worsening), are always stated as compared to status before intervention, and will be completed by the patients every 2 weeks throughout the follow-up period ***(appendix E)***.

A ”symptom score” for each of the four main symptom categories ”Fatigue”, ”Cognitive symptoms”, ”Pain” and ”Other symptoms” expresses the mean score for the symptoms under each category recorded every two weeks on the self-report form.

The Fatigue score is recorded every two weeks as the mean score for the following four symptoms: Fatigue, Post-exertional malaise, Need for rest and Daily function.

The Cognitive score is recorded every two weeks as the mean score for the following three symptoms: Concentration difficulties, Memory problems, and Mental tiredness.

The Pain score is recorded every two weeks as the mean score for the following symptoms: Muscle pain, Joint pain, Headache and Skin pain, provided that the patient actually suffers from the specific pain symptom (preregistration at baseline ≥ 5, scale 1-10).

For the category ”Other symptoms” score, changes are recorded for the two symptoms perceived as characteristic for the individual patient’s ME/CFS, out of the five symptoms in this category with the highest score on the preregistration form at baseline.

An average for each symptom score (Fatigue score, Cognitive score, Pain score, ”Other symptoms” score) for the time intervals 0-4 months, 4-8 months, 8-12 months, 12-16 months, 16-20 months, 20-24 months during the follow-up period [10].
The mean Fatigue score for the specified intervals is analysed by General Linear Model (GLM) for repeated measures, which can be used to compare longitudinal change in Fatigue score for rituximab group and placebo group during 24 months follow-up. This analysis constitutes the primary endpoint.

The mean Fatigue score over the last 4 months will be recorded at 8, 12, 16, 20 and 24 months. The difference in changes in Fatigue score from baseline to 16-20 months (mean) between rituximab group and placebo group constitutes a secondary endpoint.

”Total function level"

Changes in self-reported symptom scores compared to baseline will be relative, as significant change (i.e. value 6, scale 0-6) will be perceived differently by a patient who is somewhat active and on their feet prior to intervention, and a patient who is seriously ill and mainly bedridden prior to intervention.
Therefore, the patients will estimate their «total function level» every two weeks, as a percentage of a totally healthy state before symptom debut (which corresponds to 100 %), according to the sheet of examples in the patient folder ***(appendix D).***

Mean ”Total function level ” during intervals 0-4 months, 4-8 months, 8-12 months, 12-16 months, 16-20 months, 20-24 months, will be used as variables for GLM for repeated measures to compare changes over time by intervention group for self-reported “Total function level” during 24 month follow-up.
Mean "Total function level" over the last 4 months is recorded at 8, 12, 16, 20 and 24 months. The difference in changes in Fatigue score from baseline to 16-20 months (mean) between rituximab group and placebo group constitutes a secondary endpoint.

The equivalent self-report form has been used in the studies KTS-1-2008 [10] and the recently completed study KTS-2-2010, where both doctors and patients agreed that the form was able to capture symptom change over time.

Self-reported perception of group allocation at 6 weeks

6 weeks after the first intervention the patients will answer a question regarding which group the patient believes he/she has been allocated to (***appendix E***). The options are as follows: The patient believes he/she has received active medicine/rituximab, the patient believes he/she has received placebo/saline, or the patient has no opinion as to the intervention received.

Self-reported overall assessment of development during 24 months follow-up

At 24 months the patient will record in the self-report form any changes in the overall ME/CFS symptoms over the 24 months follow-up period. This registration should not

be based on status at 24 months, but reflect the overall development during the study, including the degree and duration of any change. There are four categories: worsening, mainly unchanged, moderate improvement, major improvement (***appendix E***).

SF-36 questionnaire on health, and analysis

-The Short Form 36 (SF-36) questionnaire on health will be completed by patients before intervention and at 3, 6, 9, 12, 15, 18, 21 and 24 months ***(appendix F1)***.
At each visit a completed SF-36 questionnaire is handed in (no copy in patient folder).
SF-36 v1.2 is a generic (diagnosis-independent) form which is widely evaluated [33,34]. We use a Norwegian validated translation [35].
In the SF-36 questionnaire for follow-up at 12, 15, 18, 21 and 24 months, question 2: “Compared to one year ago, how would you rate your health in general now?” has been replaced with: “Compared to before the start of the study, how would you rate your health in general now?”

The SF-36 is analysed using a standardized SPSS syntax file, where the results for ”Physical health summary score” and ”Mental health summary score” are interpreted using norm-based scoring (population mean = 50), and the results for the eight SF-36 subdimensions can be expressed either as ”raw scores” (scale 0-100) or as norm-based scores (US 1998).

The SF-36 ”Physical health summary score” (norm-based) and the SF-36 subdimension ”Physical Function” expressed as a raw score (scale 0-100), plus the mean SF-36 raw scores for the five subdimensions (”Physical Function”, ”Bodily Pain”, ”Vitality”, ”Social Function” and ”General Health”, scale 0-100), at 0, 3, 6, 9, 12, 15, 18, 21, 24 months, will be used for statistical analysis.
These three variables will be analysed (using GLM for repeated measures) for comparisons between rituximab group and placebo group, of longitudinal changes in ”Physical health summary score”, ”Physical Function” and ”Mean score for five SF-36 subdimensions” during 24 months follow-up.

Moreover, changes in the same SF-36 scores (”Physical health summary score”, ”Physical Function” raw score and mean score for five subdimensions (PF, BP, GH, V, SF) from baseline to each of the time points12, 15, 18, 21 and 24 months follow-up. Difference in change of these three SF-36 scores from baseline to 18 months follow-up between rituximab and placebo groups constitute a secondary endpoint.

Fatigue Severity Scale

The Fatigue Severity Scale (FSS) form consists of 9 questions related to fatigue, where each item is scored from 1 (completely disagree) to 7 (completely agree), and the patient’s FSS score equals the mean score for the 9 items. FSS has been used in a variety of studies on ME/CFS patients (***appendix F2***).
The patients will complete the FSS at baseline and after 6, 12, 18 and 24 months follow-up.
At each visit the completed form is handed in and stored in the patient case file (no copy in patient folder). The FSS score during follow-up will be the subject of statistical analysis for comparisons between the rituximab and placebo group.

Difference in change in FSS scores between the rituximab and placebo group from baseline to 18 months follow-up constitutes a secondary endpoint.

Doctor's registration at baseline and follow-up, including toxicity

At baseline assessment, the doctor will assess the ME/CFS severity for each patient: Mild, Mild/Moderate, Moderate, Moderate/Severe, or Severe (***appendix G***).
The assessing physician will assess and record individual patient symptoms at baseline pre-intervention (scale 1-10).
Registration of symptom change at follow-up (scale 0-6) and toxicity assessment will be performed at visits at 3, 6, 9, 12, 15, 18, 21 and 24 months and recorded on the form ***(appendix G)***.
Patient medicine records will be registered. Any dietary supplements must be recorded, and during the study period the patients may not start taking any new supplements without consulting a study doctor.
Any side effects including infections are recorded. Each visit must be documented in the patient's electronic journal.

Sensewear armbands for recording activity level at home

The patients' level of physical activity measured with a Sensewear armband for 7 consecutive days will be recorded after inclusion in the study, and before start of intervention with rituximab or placebo. Activity registration with a Sensewear armband must take place before ergospirometry (two consecutive days).
The registration is repeated for 7 consecutive days during the time interval 17-21 months after start of intervention.
The patients will be encouraged to maintain physical activity corresponding to their clinical condition during the registration period. Sensewear armbands are validated and used to assess physical activity in patients with rheumatoid arthritis, and is considered suitable for monitoring changes in patient physical activity after interventions [36,37].
The Sensewear armband will be distributed with a letter of information to all study participants from the trial site at Haukeland University Hospital with a return envelope for return to HUS after 7 days registration. The information on all armbands will be downloaded and analysed at the Oncology Dept., HUS.

Level of physical activity measured with a Sensewear armband for 7 consecutive days will be recorded before intervention and at 17-21 months after intervention.
Based on the preliminary analyses of Sensewear data for ME/CFS patients in the completed clinical study KTS-2-2010, the change in mean number of steps per 24 hours, the change in mean number of steps per 24 hours, the change in mean duration (≥ 3,5 METs), and the maximum duration of moderate activity level (≥ 3,5 METs) per 24 hrs are recorded.

Other variables from the Sensewear armbands include:
Mean per 24 hours for: total energy expenditure, active energy expenditure (>1,6 METs), mean METs, time for physical activity (expressed as sedentary <1,6 METs, light activity 1,6-3,0 METs, moderate activity 3,1-6,0 METs, vigorous activity >6,0 METs), total duration of physical activity (>1,6 METs), time lying down, sleep duration, duration on-body per 24 hrs (armband must in principle be worn at all times except during bath/shower).

Changes from baseline to 17-21 months, for rituximab and placebo groups will be analysed.

The patients will not receive information about the analysis result from the Sensewear activity recording at baseline or during 17-21 months follow-up, until the randomisation code has been broken (after the last included patient has completed 24 months follow-up).

MONITORING

The study will be monitored continually by external monitor Ingunn H. Anundskås, Innovest AS (Bergen). Investigators will allow direct access to source data including entries in the electronic patient journal, during monitoring, audit or inspection from the Norwegian Medicines Agency (NoMA).

INFUSION, RITUXIMAB OR PLACEBO

The patients will have completed assessment/workup, signed the written consent form and had all relevant tests and samples done before intervention. The patients can be formally admitted to an inpatient or outpatient department on the morning they are to receive an intravenous infusion of rituximab or placebo, or attend a suitable outpatient clinic, according to the established routines at every trial site.

The patients will receive induction treatment with rituximab intravenously, dose 500 mg/m^2, max 1000 mg, diluted in NaCl 0,9 % to a concentration of 2 mg/ml, or placebo, two infusions with two weeks interval. The second infusion will be administered 10-18 days after the first infusion (same dose). On the day of infusion all patients will receive the following premedication: Zyrtec (Cetirizine) 10 mg x 1 po, Paracetamol 1 g x 1 po, and Dexamethasone 8 mg x 1 po.

Maintenance infusions after 3, 6, 9 and 12 months will also be administered intravenously, with rituximab 500 mg (fixed dose) or placebo, with the same premedication and infusion speed as the induction infusions. For maintenance infusions we will allow a flexibility of +/- 10 days relative to the time lapsed after intervention start date, in order to facilitate the practical execution of treatments ***(appendix C)***.

The intravenous infusion speed will not follow the guidelines for rituximab infusions in lymphoma treatment, as the experience from our completed phase II study (KTS-2-2010) indicate that rituximab infusions may aggravate the ME/CFS symptoms in some patients shortly after the infusion, and that such reactions seem to be moderated by a lower infusion speed. Therefore, the set infusion speed will be a minimum of 4 hours for all 6 treatments.
Rituximab is usually diluted in NaCl 0.9% to a concentration of approx. 2 mg/ml and administered at an initial speed of 12 ml/hour for 30 mins, then 25 ml/hour for 30 mins, then 50 ml/hour for 30 mins, then 75 ml/hour for 30 mins, then 100 ml/hour for 30 mins, then 125 ml/hour for 30 mins, then 150 ml/hour for the remainder of the

infusion. A nurse shall be present and monitor blood pressure, heart rate and saturation as specified in the protocol (***appendix C***).

Rituximab diluted in saline can sometimes form a little foam during dilution, transport or in the drip chamber during administration, thus it can be recognized by the nurse responsible for administration. The hospital pharmacy at Haukeland University Hospital has developed a placebo solution with a visual resemblance to rituximab (Mabthera®), which consists of saline solution with a low concentration of human albumin. After visual inspection of several test solutions, the conclusion is that NaCl 0.9% with added Human Albumin (Flexbumin®) to a concentration of 0.4 mg/ml resembles the rituximab solution in colour, viscosity and tendency to form surface foam. Specific documentation for the placebo solution has been forwarded to the Norwegian Medicines Agency. The placebo solution is produced aseptically for each individual patient immediately before administration by the hospital pharmacies which are responsible for providing study medicine to the trial sites.

The hospital pharmacies responsible for the preparation of infusion bags are also responsible for maintaining drug accountability records for each study patient. At Notodden hospital, which has no hospital pharmacy, the hospital pharmacy at Skien hospital will be responsible for preparation, labelling, transport of study drug to site, as well as the drug accountability records. The temperature during transport from Skien to Notodden will be monitored.

Blinding will follow the GCP guidelines. Nurses responsible for administering the infusions shall not discuss the contents of the infusion bag with the patients. During the completed study [10] five patients in the rituximab group and four patients in the placebo group experienced some discomfort during the first 24 hours after the first infusion, thus the occurrence of symptoms and discomfort during or after treatment is not necessarily indicative of which intervention group the patient has been allocated to.

-Patients included at HUS will receive infusions at the Oncology Department’s outpatient clinic or inpatient ward.
-At Notodden Hospital infusions are administered at the chemotherapy unit at the Dept. of Medicine.
-Patients included at OUS will receive infusions at the Medical Clinic, OUS Ullevål.
-At St. Olav’s Hospital the infusions are administered in cooperation with the outpatient clinic at the Dept. of Oncology.
-At UNN the infusions are administered at the Division of Rehabilitation Services.

The trial sites are responsible for treatment of any medical complications during infusion such as allergic reactions, and during the follow-up period with the option of extra assessments and laboratory tests, as well as emergency admission in a suitable hospital ward if this should be required (e.g. for late onset neutropenia, if the patient has an elevated temperature).

EXAMINATIONS AND REGISTRATION IN THE STUDY

EXAMINATIONS AND REGISTRATION AFTER SIGNED INFORMED CONSENT, BEFORE INTERVENTION (BASELINE)

Clinical assessment: The doctor assessing the patient will check the inclusion and exclusion criteria and decide whether there is any need for supplementary testing. If an MRI of the brain has been performed during the last 5 years and no new symptoms give reason to suspect CNS pathology, a new MRI will not be necessary.
The assessment will involve exclusion of other medical conditions which may cause considerable fatigue such as: hypothyreosis, adrenal insufficiency, malignity, chronic infections, lung disease, angina pectoris, heart failure, kidney failure, liver disease, other neurological diseases (multiple sclerosis, brain tumours or cerebrovascular disease), endogenous depression or other psychiatric conditions associated with fatigue.

The doctor will assess the ME/CFS symptomatology and record the severity of the symptoms (Mild, Mild/Moderate, Moderate, Moderate/Severe, and Severe) on the relevant form ***(appendix G)***.

-The patient will complete several questionnaires for assessment of symptoms; the modified DePaul questionnaire ***(appendix B)***, the SF-36 quality of life questionnaire (***appendix F1***), the Fatigue Severity Scale (FSS) questionnaire (***appendix F2***) and the Hospital Anxiety and Depression Scale (HADS) questionnaire (***appendix F3***).
-The modified DePaul, SF-36, FSS and HADS questionnaires shall be handed in after completion and will be stored in the patient's case file at the trial site.
-The patients will also complete the self-report form for symptoms at baseline (scale 1-10) and the "Total function level" (0-100 %, in accordance with the instructions in the study folder (***appendix D***)). A copy of the self-report form is stored in the case file, while the original stays in the patient's study folder.

Any blood tests featured under Immunology, Endocrinology and/or Microbiology (see below) which have already been performed over the previous 6 months, need not be repeated at baseline.

Laboratory tests

-Hb, ESR, WBC differential, Platelet count, MCV.
-Ferritin, Fe, TIBC, Vitamin B12, Folate, Na, K, Ca, Mg, Phosphate, Glucose.
-Creatinine, Urea, Urate, Triglycerides, Total Cholesterol, HDL and LDL Cholesterol, Homocysteine, Methylmalonic Acid, 25-hydroxy-vitamin D.
-ALT, ALP, GGT, Bilirubin.
-CRP, Albumin, Total Protein, INR.
-HCG for women of childbearing age.

Immunology

-Serum Protein Electrophoresis, Quantitative Immunoglobulins with IgG, IgG subclasses, IgM, IgA.

-Immunophenotyping of mononuclear cells in peripheral blood (lymphocyte quantification). This sample must be sent to the Dept. of Transfusion medicine and Immunology at HUS, att.: Head of Dept. prof. Einar K. Kristoffersen, labelled ”Rituximab study”) ***(appendix I)***.
-tTGA (Celiac Disease Test), Antinuclear Antibody Test, anti-CCP, Thyroid Antibodies (Anti-TPO), Cardiolipin antibodies.
-Complement (C3, C4) and Complement Function (CH50).

Endocrinology
-FT4, TSH, Prolactin, Cortisol/ACTH.

Microbiology
-Serology for EBV, CMV, HSV, VZV, Parvovirus B19, Borrelia, HIV, Hepatitis serology (HBV, HCV). Quantiferon-TB test.

Biobank blood tests
(see Appendix I)
-Subject to patient consent, a 4 mm punch biopsy from macroscopically normal skin/underlying tissue laterally on the upper thigh or a true-cut needle biopsy (G14 or G16) from the vastus lateralis (laterally on the thigh) may be performed (under local anaesthetic) before intervention and at 17-21 months follow-up (optional).
-Subject to patient consent, a sample of cerebrospinal fluid may be collected and frozen at -80^{0}C, before intervention and at 17-21 months follow-up (optional).

Sensewear armband for activity registration for 7 consecutive days
The Sensewear armband will be mailed to all study participants from the trial site at HUS, along with a letter of instructions and a stamped return envelope to be used after the 7 day registration period.

BASELINE EXAMINATIONS FOR PATIENTS PARTICIPATING IN SUBSTUDIES

Substudy: Endothelial function in ME/CFS
For patients included at the Haukeland University Hospital and Notodden Hospital, an endothelial function test (Flow Mediated Dilation, FMD) will be performed before intervention and repeated once at 17-21 months after intervention.
For patients included at HUS, a test for microvascular endothelial dysfunction using Periflux-5000 will be performed before intervention and repeated once at 17-21 months after intervention ***(see separate section of protocol)***.

Substudy: Ergospirometry in ME/CFS
This substudy will include patients with a mild, mild/moderate or moderate degree of ME/CFS, where the patient and the doctor agree that the patient is capable of performing an ergospirometry test. The test must be performed after clinical assessment, all blood samples, Sensewear activity registration, endothelial function testing and any optional samples such as biopsies and spinal fluid have been collected.
A minimum of three weeks must pass from the completed ergospirometry to the start of intervention (rituximab or placebo), as the patient’s condition may deteriorate temporarily after a physical exercise test. Ergospirometry is performed with an

identical setup on two consecutive days, before intervention and again at 17-21 months follow-up ***(see separate section of protocol).***
The ergospirometry test will be performed up to maximum load. The exercise capacity (expressed in Watt) and oxygen uptake at maximum load, and at anaerobic threshold, will be measured. We will use an ergometer bike with pre-programmed load.
For ME/CFS patients who are incapable of reaching the anaerobic threshold and terminate the test due to fatigue or other symptoms, the workload (Watt) and oxygen uptake at termination, and reason for termination, will be recorded.
If the patient consents, a needle biopsy performed under local anaesthetic (true-cut, G16 or G14) from the vastus lateralis is optional. Biopsies may be performed before the first ergospirometry test on day 1 and 2-4 hours after completed ergospirometry on day 2.

Substudy: Irritable Bowel Syndrome and functional dyspepsia in ME/CFS

Patients included in the main clinical study at HUS who suffer considerable symptoms from the gastrointestinal region, will be invited to take part in an extended gastrointestinal assessment. The examinations will be performed at baseline, and again at 17-21 months after intervention start date ***(see separate section of protocol)***.
In the substudy, the following validated questionnaires will be used: ROMA III, EPQ-N and the specific IBS symptom questionnaire IBS-SSS. The antral and proximal gastrointestinal motility will be assessed using ultrasound after a standardised meal (soup).
The effect of a meal on the gastrocolic reflex will also be evaluated by assessing the motility of the smooth muscle of the sigmoideum. The substudy patients will also be offered an (optional) examination using gastroduodenoscopy, with a biopsy from the duodenum for immunohistochemistry and inflammation markers. These examinations will be performed at baseline and repeated at 17-21 months after intervention start date.

EXAMINATIONS AND REGISTRATION AT 3, 6, 9, 15, 18 AND 21 MND FOLLOW-UP

Clinical assessment with registration and entry in medical records

At each visit the doctor will perform a clinical assessment and dictate an entry in the patient's electronic records. The doctor will record any symptom change, (scale 0-6) on a separate form.
The patient's completed self-report form (symptom change (scale 0-6) and total function level (scale 0-100), recorded every two weeks) for the relevant time period is copied and stored in the patient's case file. The original form stays in the patient's study folder.
The SF-36 quality of life questionnaire for the relevant visit is handed in and stored in the patient's case file (no copy in patient folder).
FSS for the relevant visit is handed in and stored in the patient's case file (no copy in patient folder), at 6 and 18 months (as well as 0, 12 and 24 months).

Laboratory tests
-Hb, ESR, WBC differential, Platelet count, Na, K, Ca, Phosphate, Glucose, Creatinine, Urea, Urate, ALT, ALP, GGT, LD, Bilirubin, CRP, Albumin, Total Protein.

Immunology
Immunophenotyping of mononuclear cells in peripheral blood (lymphocyte quantification) at 21 months is sent to the Dept. of Transfusion medicine and Immunology at HUS, att.: Head of Dept. prof. Einar K. Kristoffersen, labelled ”Rituximab study”) ***(appendix I)***.

Blood samples for biobank
Blood samples for the biobank are collected at 6 and 18 months ***(appendix I).***

BETWEEN 17 AND 21 MONTHS (ALL PATIENTS)

Sensewear armbands for activity registration for 7 consecutive days
The Sensewear armbands will be mailed to all study participants from the trial site at HUS, along with a letter of instructions and a stamped return envelope. Download and analysis of all Sensewear data takes place at the Oncology Dept. at HUS.

BETWEEN 17 AND 21 MONTHS, FOR SUBSTUDY PATIENTS
See separate chapter for substudies in final section of protocol
Substudy: Endothelial function in ME/CFS.
Substudy: Ergospirometry in ME/CFS.
Substudy: Irritable Bowel Syndrome and functional dyspepsia in ME/CFS.

EXAMINATIONS AND REGISTRATION AT 12 AND 24 MONTHS FOLLOW-UP

Clinical assessment with registration and entry in medical records
At each visit the doctor will perform a clinical assessment and dictate an entry in the patient’s electronic records. The doctor will record any symptom change, (scale 0-6) on a separate form.
The patient’s completed self-report form (symptom change (scale 0-6) and total function level (scale 0-100), recorded every two weeks) for the relevant time period is copied and stored in the patient’s case file. The original form stays in the patient’s study folder.
The SF-36 quality of life questionnaire for the relevant visit is handed in and stored in the patient’s case file (no copy in patient folder).
At 24 months follow-up, the patient shall record his/her experience of any changes to the ME/CFS symptoms overall throughout the 24 months follow-up period (see below).

Laboratory tests
-Hb, ESR, WBC differential, Platelet count, Na, K, Ca, Phosphate, Glucose, Creatinine, Urea, Urate, ALT, ALP, GGT, LD, Bilirubin, CRP, Albumin, Total Protein.

Immunology
-Quantitative Immunoglobulins with IgG, IgM, IgA.
Immunophenotyping of mononuclear cells in peripheral blood (lymphocyte quantification) at 24 months is sent to the Dept. of Transfusion medicine and Immunology at HUS, att.: Head of Dept. prof. Einar K. Kristoffersen, labelled ”Rituximab study”) ***(appendix I)***.

Blood samples for biobank
(See appendix I).

Final visit at 24 months
At the visit after 24 months, the patient shall record in the self-report form ***(appendix E)*** his/her experience of changes, if any, to the overall ME/CFS symptom pattern throughout the 24 months of follow-up. This registration should not be influenced by the patient’s status at the 24 month visit, but rather reflect the overall development throughout the study period.

At 24 months, the patient shall record one of the following categories:
1. Worsening of ME/CFS symptoms during the 24 month study period
2. No significant change in ME/CFS symptoms during the 24 month study period, beyond habitual symptom variation
3. Moderate improvement of ME/CFS symptoms during the 24 month study period
4. Major improvement of ME/CFS symptoms during the 24 month study period.

Whether a patient should record any improvement as moderate or major, will depend on the degree and duration of the improvement, but does not depend on the patient’s continued response at 24 months.
The difference in distribution in these response categories in rituximab and placebo groups is not an endpoint in the study, but will be recorded and reported.

BIOBANK FOR BIOLOGICAL STUDIES AND IMMUNOPHENOTYPING
Blood samples for all patients who have been included in clinical studies of ME/CFS at the Dept. of Oncology, HUS, have been collected at baseline and throughout follow-up and stored in a biobank. We will systematically extend the existing approved biobank at HUS with biological material from patients included in this study, before intervention and throughout 24 months of follow-up.

Existing biobank: *Medical intervention for chronic fatigue syndrome*. Responsible: Olav Mella. Approved by REK prior to May 5th, 2009. Project number 5.2008.67.

Case/file number 2998000657-9/MRO/400. Form number in previous database notification 2219. EudraCT number 2007-007973-22. Approved by the Norwegian Directorate of Health. Physical location: Department of Oncology and Medical Physics, Haukeland University Hospital. For each project and clinical study regarding ME/CFS at the Dept. of Oncology at Haukeland University Hospital during 2009-2014, applications to the Regional Ethical Committee have included applications for extending the existing biobank.

The biobank will be a starting point for further research into the pathogenesis of ME/CFS. The mechanisms behind the disease must be charted, and a specific and sensitive biomarker is greatly needed.
In addition to laboratory tests as specified above, before treatment and at follow-up after 3, 6, 9, 12, 15, 18, 21 and 24 months, blood samples for biobank and research will be collected at baseline and after 6, 12, 18 and 24 months ((informed consent obtained before inclusion). Complete sets of pre-labelled blood collection tubes will be sent from HUS to all trial sites. The samples can be stored temporarily at a local biobank at each trial site, and forwarded in larger consignments to the central biobank at the Dept. of Oncology, Haukeland University Hospital (***appendix I***).

At baseline and after visits at 21 and 24 months an EDTA tube (3 ml) of whole blood shall also be sent to the Dept. of Transfusion Medicine and Immunology at HUS, att.: Head of Dept. Prof. Einar K. Kristoffersen, for immunophenotyping of lymphocyte populations in peripheral blood. This sample must be stored and shipped at room temperature, and must arrive at HUS within 3 days. The tube must be labelled with ”Rituximab study”, the patient’s study ID number and number of months after intervention ***(appendix I)***. The immunophenotyping analysis results will be stored at the Dept. of Transfusion Medicine and Immunology until the last included patient has completed at least 24 months follow-up, the study is closed and the randomisation code broken.

Additional samples are optional, and may include a punch biopsy from skin/underlying tissue on the thigh for formalin fixation, a true-cut needle biopsy from the lateral thigh muscle for protein purification, RNA purification and paraffin-embedding and/or cerebrospinal fluid for freezing, before intervention and possibly repeated at 17-21 months.

PATIENT WITHDRAWAL DURING STUDY

The patients will be informed verbally and in the written patient information that they may withdraw from the study at any time, without having to state the reason for their decision. The medical reasons for withdrawing a patient from the study may be serious events such as severe allergic reactions during or short time after the infusion. Patients who withdraw from the study due to intercurrent or other diseases or any other reason will be followed by their general practitioner according to usual ME/CFS guidelines. If possible, we will attempt to obtain toxicity data from patients who have withdrawn during the study.
If a patient is included in the study, but withdraws before intervention or for other reasons does not receive the rituximab intervention, this patient may be replaced by

another patient. In the sample power analysis a withdrawal rate of approx. 5 % is estimated, i.e. an estimated 8 patients will not receive the intervention or contribute to the analyses (missing data).

ADVERSE EVENTS, SAFETY BOARD, SIDE EFFECTS

The study management at each trial site is responsible for follow-up on any reported side effects during the study period. The local PI will report any serious adverse events to the central study management at HUS, who are responsible for further reporting to the Regional Ethical Committee and the Norwegian Medicines Agency (NOMA).

AEs (Adverse Events) will be reported in the eCRF.

SAEs (Serious Adverse Events) are defined as Adverse Events which are deadly or life-threatening, result in hospitalization (initial or prolonged), lasting or significant disability or incapacity or a congenital anomaly or birth defect, or is considered serious for other reasons.
SAEs must be reported on a separate form ***(appendix H)*** and sent to sponsor (Haukeland University Hospital, the Dept. of Oncology and Medical Physics, att.: Head of Dept. Olav Mella) within 24 hours after the trial site being notified of the event.

A SUSAR (Suspected Unexpected Serious Adverse Reaction), is defined an SAE which is suspected related to the trial medication, and is also unexpected.
SUSAR must be reported to NOMA according to the following guidelines: all deadly or life-threatening SUSARs must be reported to NOMA immediately and within 7 days of sponsor being notified of the event. Other SUSARs (unblinded) must be reported to NOMA within 15 days.

Only SUSARs will be reported as an individual report. The responsibility for reporting of SUSARs to NOMA and for the distribution of relevant information to the remaining PIs, lies with the central study management at the Oncology department at HUS.

The remaining adverse events will be reported in a combined final report. The central study management at HUS is responsible for annual reporting of serious adverse events to NOMA.

Changes in ME/CFS symptoms during the follow-up period and temporary worsening of symptoms during the weeks immediately following the rituximab infusions will not be recorded as Adverse Events. Peroral antibiotic treatments over a shorter period of time for otherwise uncomplicated infections (URI, lower UTI) are recorded and reported in the combined final report.

Safety Board and side effects

The safety aspect of the study will be supervised by a Safety Board. Members are Prof. Olav Dahl, Dept. of Oncology, Haukeland University Hospital (Chairman),

Prof. Ola Didrik Saugstad, Dept. of Paediatrics, Rikshospitalet OUS and Senior Consultant Unn Merete Fagerli, Ph.D., Dept. of Oncology, St. Olav's Hospital.

The Safety Board will monitor Adverse Events (AE) and Serious Adverse Events (SAE) in the study. Members of the Safety Board will be given access to information on the intervention administered to a specific patient (unblinding) after contacting the hospital pharmacy at the relevant trial site, if a medical event should occur where knowledge of the patient's B-lymphocyte depletion status is required. The responsibility for reporting of unblinded SUSARs is delegated to the Chairman of the Safety Board, prof. Olav Dahl.
If the members of the Safety Board are unavailable, and the treatment of a medical event requires knowledge of which intervention group the patient belongs to, the study management represented by Olav Mella or Øystein Fluge shall be given access to information on the specific patient's intervention group after contacting the relevant hospital pharmacy.

Members of the Safety Board will not take part in the clinical assessment of patients.

The study is double-blind and placebo controlled, and thus there are no plans for an interim analysis of the response data or side effects. Possible side effects will be monitored continually according to protocol. The safety profile of rituximab in several patient populations is well documented. However, the experience of rituximab treatment in the subject population is limited. In the event of SAEs, the study management in collaboration with the Safety Board will consider the need for unblinding in order to establish or rule out association with rituximab and, if relevant, characterise the SAE as a SUSAR. Should several SUSARs occur, the study management and Safety Board will consider discontinuing the inclusion of patients in the trial.

All safety data compiled in the study will be submitted as part of the secondary parameters for analysis, which will be reported in the final study results and end of study report.

Work-up before inclusion should exclude patients with suspected immunodeficiency disorders or ongoing active and relevant viral infections. However, the cause of ME/CFS is as yet unknown, and we cannot exclude the possibility that some patients may suffer an ongoing active viral infection which is undetectable at the work-up, and where B-cell depletion could theoretically cause a clinical deterioration.
In our ongoing trial with rituximab induction and maintenance treatment, although we have noted that some patients (25%) experience a temporary worsening in ME/CFS symptoms up to 6-8 weeks after rituximab infusion, we have only observed one patient (in the KTS-2-2010 trial) who has reported a lasting decline in function compared to status before intervention. This patient was also in gradual decline during the year before inclusion.
All other patients in the pilot series or in the trials KTS-1-2008, KTS-2-2010, or KTS-3-2010 (a total of approx. 50 patients treated with rituximab) have experienced either a clinical response or a mainly unchanged status (non-responders) after intervention, throughout a follow-up period of up to five years.

We are aware of a case report describing a patient with severe ME/CFS treated outside clinical studies in Germany, who experienced a lasting deterioration in his symptoms for at least half a year after rituximab intervention. Diagnostics pointed to circulating IgM against rituximab as a possible cause of the deterioration due to formation of immune complexes.
In the KTS-2-2010 trial, one patient had an allergic reaction with shortness of breath immediately following the first infusion. There were no further complications, but the patient was not given further rituximab infusions.
Two patients in the KTS-2-2010 trial experienced one episode of "Late onset neutropenia" (LON), a maturation arrest in neutrophilic granulocytes which can be seen in 5-10 % of patients receiving rituximab treatment for lymphomas, often observed when B cells start regenerating in the bone marrow. Both patients experienced uncomplicated LON for the duration of 4 to 5 days. Both patients experienced some drowsiness (different to their habitual ME/CFS condition), a slightly reduced general condition and both reported slightly tender gums. It is important to be aware of this possible side-effect, as the neutropenic patient can be susceptible to serious infections, especially if neutrophils are $< 0,3 \times 10^6$/mL. In this case, the patient must be admitted to a suitable hospital department.
Patients will receive information on the safety aspects orally and in the letter of information.

ETHICAL ASPECTS

ME/CFS is a serious affliction involving considerable suffering for the patient and great distress for relatives as well. No standardized, established medical treatment exists. Many young people are affected by the condition, with an estimated prevalence of approx. 0.1-0.2 % of the population, i.e. between 5,000 and 10,000 patients in Norway and approx. 10,000,000 patients worldwide.
B-cell depletion using rituximab is still an experimental intervention. The risk involved in rituximab treatment is low, but not negligible. In our opinion, the risk is acceptable considering the possible health gain for patients. ME/CFS sufferers are a large group including a young population unable to attend work or studies, and suffering considerable symptoms. If the study confirms that B cell depletion is associated with a clear response in ME/CFS patients, this could have a great impact on the lives of many patients.

Therapeutic B cell depletion with rituximab is considered a safe treatment in patients with lymphomas and autoimmune disorders. Nevertheless serious side effects do occur, albeit very rarely. The most serious side-effect is progressive multifocal leukoencephalopathy (PML) caused by reactivation of the JC virus with brain infection. PML is often deadly, and occurs in approx. 1 of 25.000 arthritis patients treated with rituximab, usually in combination with other immunosuppressive agents [38].
Another serious side-effect is the possible reactivation of hepatitis, infections or interstitial pneumonitis. Sometimes allergic reactions occur during and immediately after infusion. One patient had to withdraw from the KTS-2-2010 trial due to a probable allergic respiratory reaction with temporary shortness of breath towards the end of the first rituximab infusion. Pulmonary function testing with chest x-ray,

spirometry and gas exchange calculation showed normal function after one week. The risk of such side-effects is small. In the published study [10] we have not observed unexpected or serious toxicity, and no serious infections. Two patients, both in the rituximab group, had a transient flare-up of pre-existing psoriasis, which could be a side-effect. In the ongoing phase II trial, two patients have experienced an allergic reaction; two have had recurring upper airway infections and two have had an episode of late onset neutropenia lasting 5 days without complications. While the side effect profile for rituximab in lymphomas (including maintenance treatment) and in other autoimmune diseases is well known [12], the toxicity of rituximab in ME/CFS is still largely unknown.

In the ongoing open phase II study with rituximab maintenance treatment we find that some patients (25%), despite a clear clinical response, experience a temporary deterioration in ME/CFS symptoms lasting for several weeks after a rituximab infusion. Out of the 28 patients in KTS-2-2010 who received rituximab induction and maintenance (6 infusions in total), three patients reported a significant worsening and four patients a moderate worsening of symptoms either directly following, or during the first days after infusion.
A case report exists regarding a significant worsening in ME/CFS symptoms lasting for at least 6 months after rituximab treatment outside a clinical study, where immune complex formation was considered the most probable cause of ME/CFS symptom deterioration.

The placebo solution contains a small amount of albumin, thus there is a possibility of infusion related reactions/sensitivity reactions. The risk is small, and the measures taken to prevent and treat such reactions for rituximab are also adequate for albumin.

The risk at B cell depletion with rituximab is small, but not negligible, and in our opinion acceptable considering the possible patient health gain. Through participation in the study, the individual participant has a chance of experiencing clinical improvement of symptoms which are frequently severe and disabling with regards to social and family life, work or studies and quality of life. The study will produce knowledge on ME/CFS. We will systematically extend the existing biobank with blood samples from the patients at baseline and 6, 12, 18 and 24 months follow-up, and further biological spin-off studies on the biobank material will attempt to shed light on the pathogenic mechanisms behind ME/CFS.
The patients will be invited to a consultation where they will receive in-depth information and a written letter of information/declaration of informed consent, and will be given due time to consider before deciding on whether or not to participate in the study.
The patients will be informed that the chance of being allocated to the rituximab group is 50 %. Patients allocated to the placebo group will be offered participation in a new study involving rituximab, if the results show that intervention with rituximab is associated with clinically significant responses in ME/CFS patients, and provided that funding can be obtained.

FUNDING

The study is investigator initiated. There is no external sponsor. The research team for ME/CFS at the Oncology Department at Haukeland University Hospital receives support from the Kavli trust, mainly for research into the pathogenic mechanisms behind ME/CFS.
The Norwegian Research Council has agreed to support the study with an amount which will largely cover the purchase of rituximab, which is the most substantial expense in the study budget.
The Ministry of Health and Care Services has earmarked NOK 2 million for 2012, 2 million for 2013 and 2 million for 2014 for this study.
A private fundraising (”MEandYou”) will contribute with more than NOK 2.8 million. The regional health trusts represented by the CEOs have expressed support for the study, and an application to the health trusts has been filed for part funding of salaries for the doctors taking part in patient assessment and treatment.
A trial-specific medical insurance will be taken out.
No financial compensation will be offered to the participants.

PUBLICATION

Co-authorship for the clinical study will be subject to participation in assessment and follow-up of patients at the trial sites. For supplementary biological analyses, physiological, neuropsychological or cognitive examinations, co-authorship in the main clinical study will depend on whether the data are used in the relevant publication. The order of authors in the publication will be decided by coordinating investigator Olav Mella. Co-authorship in substudies where data are not used in the main clinical study, such as cognitive testing, ergospirometry, endothelial function, gastrointestinal examinations and biological studies will be subject to active participation in the planning and implementation of the substudies.
The results – positive or negative – from the clinical study will be published in a reputable medical journal. Co-authorship and order of authors will comply with the Vancouver guidelines.
The study will be registered in ClinicalTrials.gov before study start date.
A final report will be submitted to the REC and NOMA.

APPLICATIONS FOR APPROVAL

Applications for approval will be sent to:
- The Regional Ethical Committee.
- The Biobank Register (extension of existing biobank).
- EudraCT.
- The Norwegian Medicines Agency.

SUBSTUDIES OF THE MAIN CLINICAL STUDY

SUBSTUDY: ENDOTHELIAL FUNCTION IN ME/CFS

Flow-mediated Dilation (FMD)

A study has shown that ME/CFS patients have an endothelial dysfunction detectable through a clinical test measuring the reactive vasodilation of the arteria brachialis after 4 to 5 min. occlusion using a blood pressure cuff (flow mediated vasodilation, FMD) and microvascular endothelial dysfunction measured by post-occlusive reactive hyperaemia (PORH) testing [15].
In collaboration with the Department of Cardiology at HUS, we have measured endothelial function using FMD in a total of 16 ME/CFS patients. Average FMD was 3.5%, and five patients had an FMD < 1%. This is in comparison to an average FMD of 8.5% measured in healthy women, using the same equipment and protocol, by the same two doctors. Only one out of 66 healthy women had an FMD < 2%. Thus our preliminary data support the findings in the above-mentioned study [15].

Endothelial dysfunction is a risk factor for cardiovascular diseases [16], and a slight to moderate reduction in FMD is also associated with autoimmune systemic diseases [17]. A slight or moderate association between FMD and depression is also described in some studies [18].

The term “endothelial function” describes the ability of the endothelium to respond with local vasodilation to external provocation. The main endothelium-dependent vasodilator is nitrogen monoxide (NO). An increase in vessel blood flow causes increased shear stress on the endothelium, affecting endothelial nitrogen monoxide synthase (eNOS) activity, which in turn stimulates production of NO from the substrate L-Arginine. Flow-mediated vasodilation of the arteria brachialis is the most common and best validated method for the assessment of endothelial function [39]. As described above, preliminary data from our analyses of FMD in ME/CFS patients show a significantly reduced FMD in these patients, and we hypothesize that this could be a key factor in the ME/CFS symptomatology.

All patients included at Haukeland University Hospital and Notodden Hospital will be offered participation in the substudy, and they will receive a separate written patient information/consent form. FMD will be performed before start of intervention and repeated between 17-21 months after start of intervention. We will record any changes in endothelium dependent vasodilation expressed as percentile change of the brachial artery diameter after 5 minutes cuff occlusion.
We wish to investigate whether any endothelial dysfunction (assessed by FMD) is related to the symptomatology or the severity of ME/CFS, and whether patients who experience a clinical response also experience an increase in FMD. The relation, if any, between FMD at baseline and symptom severity, classed as Mild, Mild/Moderate, Moderate, Moderate/Severe or Severe ME/CFS, will be recorded and analysed.
Responders and non-responders after rituximab treatment, and patients allocated to the placebo group, may be compared with regards to endothelial function and symptom change. Changes in FMD from baseline to 17-21 months follow up will be

recorded, and any differences between rituximab/placebo groups and responders/non-responders in the rituximab group will be analysed. Patients taking part in the substudy will not be informed of their FMD results (at baseline or follow-up), until the intervention code has been broken (24 months after first treatment, last patient).

The FMD assessment is performed under standardised conditions, observing the guidelines developed by The International Brachial Artery Reactivity Task Force [40]. Participants should not suffer from any known intercurrent disease, should not undergo the assessment during menstruation, and should fast for at least 8 hours before the assessment – i.e. abstain from food, fluids (except water), tobacco and medications. Any depot medications should be withheld for 24 hours. Subjects will be studied at approximately the same time of day, in a quiet and dark room maintaining a temperature of approx. 22° C. Prior to the assessment, the subject will relax in a supine position for at least ten minutes. A blood pressure cuff is placed on the right forearm. The ultrasound imaging is performed using the GE Dinged (GE Dinged, Vivid E9, GE, and Herten, Norway) system, with a multi-frequency linear probe, 6-13 MHz (M12L). The brachial artery is imaged in the longitudinal plane above the cubital fossa, and images are stored for reference. A marker pen is used to indicate the probe position on the skin. The blood pressure cuff, which is positioned proximally on the forearm, distally to the transducer position, is inflated to 200 mm Hg or at least 50 mm Hg above systolic pressure, for 5 minutes. Following deflation of the cuff, images are recorded continuously from the same area of the artery during the next 5 minutes. The diameter of the brachial artery is measured between the insides of the endothelium on the near and far walls of the artery. All measurements are performed during end diastole. Flow mediated dilation is measured at maximal dilation, and is expressed as a percentage of the baseline diameter.
After 10 minutes rest, a dose of nitroglycerine spray (0.4 mg) is administered sublingually, and images of the brachial artery are recorded continuously for another 5 minutes. The maximal diameter is measured to assess endothelial independent vasodilation.

Microvascular endothelial function

Assessments of microvascular endothelial dysfunction will be performed for patients included at the Haukeland University Hospital, at baseline and repeated during the time interval 17-21 months after intervention. The assessments will be performed at approximately the same time of day, and under the same standardised conditions as those applied to FMD assessments. Endothelial function will be estimated using a Periflux 5000 unit with laser doppler technology (Perimed, Stockholm), and we will assess post-occlusive reactive hyperaemia (PORH) in the skin, and the skin blood flow response to iontophoretic application of the neurotransmitter acetylcholine [41]. 0.18 ml acetylcholine 10 mg/ml (Miochol-E powder for intraocular solution dissolved in sterile water) is applied to the skin of the left forearm using iontophoresis, i.e. the application of a small electric current of 20 microampere/min. for 10 minutes, and changes in the skin circulation are recorded. PORH assessment is combined with the FMD assessment into one procedure. We will measure the skin circulation at baseline, inflate the blood pressure cuff to 200 mm Hg (or at least 50 mm Hg above systolic pressure) for 5 minutes, and finally, after cuff deflation, record the hyperaemia response during the first 2 minutes (expressed as area under the curve during 2 min. hyperaemia phase minus area under the curve during 2 min. baseline phase).

Patients in the rituximab group, i.e. responders and non-responders after rituximab infusions, and patients randomised to the placebo group will be compared in terms of relation between microvascular endothelial function and clinical response with symptom change. Changes in microcirculation from baseline to follow-up after 17-21 months are recorded and analysed for differences between rituximab and placebo groups and responders/non-responders in the rituximab group. Patients who participate in the substudy will not be informed of the results of microvascular endothelial function (at baseline or at follow-up after 17-21 months), until after the randomisation code has been broken (24 months after inclusion of the last patient).

As reference values are lacking in available literature, we will also invite 30 healthy controls between the ages of 18-65 (3/4 female, with no known chronic disease) to undergo the assessment of microvascular endothelial function with Periflux 5000 (PORH and iontophoretic application of acetylcholine). The purpose is to establish a reference material for analyses of microvascular endothelial function, and the controls will be subject to the same standardized conditions as outlined above. The controls will be recruited among staff and students at Haukeland University Hospital, and they will receive a separate information sheet and consent form (see appendix J2.1).

SUBSTUDY: ERGOSPIROMETRY IN ME/CFS

A main feature in ME/CFS is post-exertional malaise (PEM), which involves increased symptoms and fatigue with a prolonged restitution time even after moderate activity. A notable reduction in function capacity the day after a maximal work load, with a significant reduction in maximal oxygen consumption (VO_2 max) and reduced oxygen consumption at anaerobic threshold (AT) has been identified in ME/CFS patients [26,27,42]. This has not been observed in other conditions, and is seen as an expression of PEM, where the cause is as yet unknown.

This substudy is relevant for patients with mild, mild/moderate and moderate ME/CFS who consider themselves (and are considered by the investigator) physically capable of completing an ergospirometry test on two consecutive days. Patients included in the clinical main study at Oslo University Hospital, Haukeland University Hospital and Notodden Hospital, will be asked to participate and given a separate patient information and consent form.
The purpose of the substudy is to investigate whether any clinical response after B-cell depletion is associated with improved work capacity and oxygen consumption at maximum load and anaerobic threshold.

Maximum oxygen consumption and maximum work load, as well as oxygen consumption and work load at anaerobic threshold, will be registered. Tests are performed using a bicycle ergometer with a programmed ramp protocol with increases in wattage of either10 Watt/min, 15 Watt/min, 20 Watt/min, 25 Watt/min or 30 Watt/min, depending on clinical assessment, gender and symptom severity. Patients are expected to achieve maximum workload within 8 to 12 minutes [42]. The ergospirometry test is repeated on day two (after 20 to 28 hours) following the same protocol.

The stress test includes continuous registration of heart rate and respiratory gas exchange; oxygen uptake, carbon dioxide production and respiratory exchange ratio (RER).
The anaerobic threshold can be estimated based on these measurements of respiratory gas exchange, using one out of three methods: Respiratory Exchange Ratio (RER), "Ventilator equivalent for VO_2" (EQO_2) or the V-slope method [43-45].

Repeated measurements of lactate may also be performed, drawing capillary blood from a fingertip at baseline and at end of test, alternatively every three minutes during work load. For the purposes of lactate analyses, the centres may use Lactate Scout®, the accuracy and reproducibility of which have been tested at the central laboratory at Haukeland University Hospital.
If any ME/CFS patient fails to reach anaerobic threshold and terminates the test due to exhaustion or other symptoms, the reason for the termination and the oxygen uptake and work load (Watt) at the time of termination are recorded.

Ergospirometry tests are performed using the same protocol on two consecutive days before intervention and again during the time frame of 17 to 21 months after start intervention.
Oxygen uptake and work load (Watt) on day two, at maximum work load and anaerobic threshold will be compared to the equivalent values at 17 to months after start intervention. Changes from baseline (before intervention) to the repeated stress test at 17 to 21 months will be recorded and the difference between the rituximab and placebo groups analysed.

As the test protocol for measurements of oxygen uptake and work load at maximum work load and anaerobic threshold may differ somewhat at the different study sites, the relative change (in per cent) from baseline to the repeated test at 17 to 21 months will also be recorded and analysed for differences between the rituximab and placebo groups.

For example, a patient who at baseline records a work load of 28 watts at anaerobic threshold on day 2, and at 17-21 months follow-up records 38 watts on day 2, will register a 35.7 % (38 watts – 28 watts = 10 watts, 10 watts/28 watts) increase in work load at anaerobic threshold from baseline to 17-21 months.
Correspondingly, a patient who at baseline records a work load of 22 watts at anaerobic threshold on day 2, and at 17-21 months follow-up records 20 watts on day 2, will register a 9.1 % (38 watts – 28 watts = 10 watts, 10 watts/28 watts) decrease in work load at anaerobic threshold from baseline to 17-21 months.

Participants in the substudy will not be informed of the test results at baseline or at 17-21 month follow-up, until the randomisation code has been broken (24 months after inclusion of the last patient).

If the patient consents, a needle biopsy performed under local anaesthetic (true-cut, G16 or G14) from the vastus lateralis is optional. Biopsies may be performed before

the first ergospirometry test on day 1 and 2-4 hours after completed ergospirometry on day 2.

Stress tests may be combined with collection of blood samples before and after testing, which may be subject to analyses of cytokines in serum using ELISA and/or gene expression (mRNA) using quantitative RT-PCR from lymphocytes in peripheral blood, of sensory ion channels, adrenergic receptors and selected cytokines e.g. before stress testing and after 30 minutes, 8 hours, 24 hours and 48 hours [46]. Selected cytokines and markers for oxidative stress can be measured in peripheral blood.

Patients with mild or moderate ME/CFS can experience a temporary deterioration in symptoms after two consecutive days of ergospirometry stress testing. Only patients who can tolerate such exercise according to patients' own judgement and supported by clinical assessment, will be included in the substudy. Pilot testing and experience from other studies show that the ergospirometry stress testing of ME/CFS patients is feasible, and considering the possible scientific value of the substudy, we consider the tests ethically acceptable.

SUBSTUDY: IRRITABLE BOWEL SYNDROME AND FUNCTIONAL DYSPEPSIA IN ME/CFS

Post-infectious Irritable Bowel Syndrome (IBS) can occur after bacterial infections in which immune activation of intestinal mucosa plays an important part. The occurrence of enduring abdominal symptoms after parasitic infections is less recognized.

Among patients who were infected with the Giardia Lamblia parasite following contamination of the drinking water in Bergen in 2004, a high prevalence of ME/CFS has been reported [47]. An increased count of CD8 positive T-cells was detected in the peripheral blood of patients suffering from post-Giardia functional intestinal symptoms, while post-infectious ME/CFS cases showed low NK-cell counts in peripheral blood [48].

In an ongoing study at the department of Medicine at Haukeland University Hospital, the level of T- and B-lymphocytes in the duodenum has been investigated in patients with enduring gastro-intestinal symptoms after going through a Giardia infection in Bergen. 99 patients were included. Giardia was detected in the stool of all patients using microscopy and/or a quick antigen test, and they had been treated with metronidazole.

Due to lasting symptoms these patients were referred to an extended workup including gastroscopy, blood samples and new stool samples. They also completed a ROMA II form with abdominal symptom score (VAS for nausea, abdominal pain, bloating, diarrhoea and constipation). A main finding was that patients with post-infectious IBS and chronic giardiasis had a reduced count of CD4 positive T-lymphocytes in the lamina propria. An increased count of CD20 positive B cells in the lamina propria crypts was found in both the chronic giardiasis group and the post infectious IBS group, compared to healthy controls. The findings indicate sustained immunological activation in the duodenal mucosa after Giardia infection.

Patients with ME/CFS who are included in the main clinical study at Haukeland University Hospital and who suffer considerable symptoms from the gastrointestinal tract indicating functional dyspepsia or Irritable Bowel Syndrome (IBS), will be invited to take part in the substudy. The subjects will be patients with ME/CFS according to the Canadian criteria [8], age 18-65 years and a symptom duration of minimum two years and maximum 15 years. Patients with a mild degree of ME/CFS must have symptom duration of at least 5 years. It is estimated that symptom onset follows an infection in approx. 70 % of ME/CFS cases, and as such this substudy will not focus particularly on ME/CFS triggered by Giardia.

Patients who qualify for inclusion will be informed about the substudy at the clinical assessment for inclusion in the main study, and receive a separate patient information and consent form. The substudy is managed by Prof. Trygve Hausken and Prof. Odd Helge Gilja and Dr. Elisabeth Steinsvik at the dept. of Medicine, Haukeland University Hospital.

The purpose of the substudy is to investigate whether clinical responses in ME/CFS symptoms after B cell depletion using the monoclonal anti-CD20 antibody rituximab are associated with responses in gastrointestinal symptoms. Changes in symptoms and findings from baseline to follow-up at 17-21 months can be compared for differences between the rituximab and placebo groups, and between responders and non-responders in the rituximab group.

Patients included in the substudy will complete the following validated questionnaires: ROMA III, EPQ-N and IBS-SSS.
The motility of the antrum and the proximal stomach is assessed by ultrasonography after a standardised meal (soup). The effect of the meal on the gastrocolic reflex will also be assessed by examining the motility of the smooth muscle in the sigmoideum.

The participants will also be requested to undergo a gastroduodenoscopy, with a biopsy taken from the duodenum for immunohistochemistry and inflammation markers. These examinations are also performed both at baseline and at 17-21 month follow-up.

Participants in the substudy will not be informed of the test results at baseline or at 17-21 month follow-up, until the randomisation code has been broken (24 months after inclusion of the last patient).

REFERENCES

1. Nacul LC, Lacerda EM, Pheby D, Campion P, Molokhia M, et al. (2011) Prevalence of myalgic encephalomyelitis/chronic fatigue syndrome (ME/CFS) in three regions of England: a repeated cross-sectional study in primary care. BMC Med 9: 91.

2. Brenu EW, van Driel ML, Staines DR, Ashton KJ, Ramos SB, et al. (2011) Immunological abnormalities as potential biomarkers in Chronic Fatigue Syndrome/Myalgic Encephalomyelitis. J Transl Med 9: 81.
3. Broderick G, Fuite J, Kreitz A, Vernon SD, Klimas N, et al. (2010) A formal analysis of cytokine networks in chronic fatigue syndrome. Brain Behav Immun 24: 1209-1217.
4. Schutzer SE, Angel TE, Liu T, Schepmoes AA, Clauss TR, et al. (2011) Distinct cerebrospinal fluid proteomes differentiate post-treatment lyme disease from chronic fatigue syndrome. PLoS One 6: e17287.
5. Biswal B, Kunwar P, Natelson BH (2011) Cerebral blood flow is reduced in chronic fatigue syndrome as assessed by arterial spin labeling. J Neurol Sci 301: 9-11.
6. Duffy FH, McAnulty GB, McCreary MC, Cuchural GJ, Komaroff AL (2011) EEG spectral coherence data distinguish chronic fatigue syndrome patients from healthy controls and depressed patients - A case control study. BMC Neurol 11: 82.
7. Chang CM, Warren JL, Engels EA (2012) Chronic fatigue syndrome and subsequent risk of cancer among elderly US adults. Cancer 118: 5929-5936.
8. Carruthers BM, Jain AK, De Meirleir KL, Peterson DL, Klimas NG, et al. (2003) Myalgic encephalomyelitis/ chronic fatigue syndrome: clinical working case definition, diagnostic and treatment protocols. J Chronic Fatigue Syndr 11: 7-36.
9. Fluge O, Mella O (2009) Clinical impact of B-cell depletion with the anti-CD20 antibody rituximab in chronic fatigue syndrome: a preliminary case series. BMC Neurol 9: 28.
10. Fluge O, Bruland O, Risa K, Storstein A, Kristoffersen EK, et al. (2011) Benefit from B-Lymphocyte Depletion Using the Anti-CD20 Antibody Rituximab in Chronic Fatigue Syndrome. A Double-Blind and Placebo-Controlled Study. PLoS One 6: e26358.
11. Fukuda K, Straus SE, Hickie I, Sharpe MC, Dobbins JG, et al. (1994) The chronic fatigue syndrome: a comprehensive approach to its definition and study. International Chronic Fatigue Syndrome Study Group. Ann Intern Med 121: 953-959.
12. Ram R, Ben-Bassat I, Shpilberg O, Polliack A, Raanani P (2009) The late adverse events of rituximab therapy-rare but there! Leuk Lymphoma 50: 1083-1095.
13. Albright F, Light K, Light A, Bateman L, Cannon-Albright LA (2011) Evidence for a heritable predisposition to Chronic Fatigue Syndrome. BMC Neurol 11: 62.
14. Kessel A, Rosner I, Toubi E (2008) Rituximab: beyond simple B cell depletion. Clin Rev Allergy Immunol 34: 74-79.
15. Newton DJ, Kennedy G, Chan KK, Lang CC, Belch JJ, et al. (2012) Large and small artery endothelial dysfunction in chronic fatigue syndrome. Int J Cardiol 154: 335-336.
16. Sitia S, Tomasoni L, Atzeni F, Ambrosio G, Cordiano C, et al. (2010) From endothelial dysfunction to atherosclerosis. Autoimmun Rev 9: 830-834.
17. Murdaca G, Colombo BM, Cagnati P, Gulli R, Spano F, et al. (2012) Endothelial dysfunction in rheumatic autoimmune diseases. Atherosclerosis 224: 309-317.
18. Cooper DC, Tomfohr LM, Milic MS, Natarajan L, Bardwell WA, et al. (2011) Depressed mood and flow-mediated dilation: a systematic review and meta-analysis. Psychosom Med 73: 360-369.
19. Garcia X, Stein F (2006) Nitric oxide. Semin Pediatr Infect Dis 17: 55-57.
20. Coleman JW (2001) Nitric oxide in immunity and inflammation. Int Immunopharmacol 1: 1397-1406.
21. Steinert JR, Chernova T, Forsythe ID (2010) Nitric oxide signaling in brain function, dysfunction, and dementia. Neuroscientist 16: 435-452.
22. Steinert JR, Robinson SW, Tong H, Haustein MD, Kopp-Scheinpflug C, et al. (2011) Nitric oxide is an activity-dependent regulator of target neuron intrinsic excitability. Neuron 71: 291-305.
23. Stefano GB, Goumon Y, Bilfinger TV, Welters ID, Cadet P (2000) Basal nitric oxide limits immune, nervous and cardiovascular excitation: human endothelia express a mu opiate receptor. Prog Neurobiol 60: 513-530.

24. Murrough JW, Mao X, Collins KA, Kelly C, Andrade G, et al. (2010) Increased ventricular lactate in chronic fatigue syndrome measured by 1H MRS imaging at 3.0 T. II: comparison with major depressive disorder. NMR Biomed 23: 643-650.
25. Shungu DC, Weiduschat N, Murrough JW, Mao X, Pillemer S, et al. (2012) Increased ventricular lactate in chronic fatigue syndrome. III. Relationships to cortical glutathione and clinical symptoms implicate oxidative stress in disorder pathophysiology. NMR Biomed 25: 1073-1087.
26. Vermeulen RC, Kurk RM, Visser FC, Sluiter W, Scholte HR (2010) Patients with chronic fatigue syndrome performed worse than controls in a controlled repeated exercise study despite a normal oxidative phosphorylation capacity. J Transl Med 8: 93.
27. Vermeulen RC, Vermeulen van Eck IW (2014) Decreased oxygen extraction during cardiopulmonary exercise test in patients with chronic fatigue syndrome. J Transl Med 12: 20.
28. Brenu EW, van Driel ML, Staines DR, Ashton KJ, Hardcastle SL, et al. (2012) Longitudinal investigation of natural killer cells and cytokines in chronic fatigue syndrome/myalgic encephalomyelitis. J Transl Med 10: 88.
29. Hellsten Y, Nyberg M, Jensen LG, Mortensen SP (2012) Vasodilator interactions in skeletal muscle blood flow regulation. J Physiol 590: 6297-6305.
30. Green DJ, Jones H, Thijssen D, Cable NT, Atkinson G (2011) Flow-mediated dilation and cardiovascular event prediction: does nitric oxide matter? Hypertension 57: 363-369.
31. Mineo C, Shaul PW (2012) Regulation of eNOS in caveolae. Adv Exp Med Biol 729: 51-62.
32. Zigmond AS, Snaith RP (1983) The hospital anxiety and depression scale. Acta Psychiatr Scand 67: 361-370.
33. Ware JE, Jr., Sherbourne CD (1992) The MOS 36-item short-form health survey (SF-36). I. Conceptual framework and item selection. Med Care 30: 473-483.
34. Myers C, Wilks D (1999) Comparison of Euroqol EQ-5D and SF-36 in patients with chronic fatigue syndrome. Qual Life Res 8: 9-16.
35. Loge JH, Kaasa S, Hjermstad MJ, Kvien TK (1998) Translation and performance of the Norwegian SF-36 Health Survey in patients with rheumatoid arthritis. I. Data quality, scaling assumptions, reliability, and construct validity. J Clin Epidemiol 51: 1069-1076.
36. Almeida GJ, Wasko MC, Jeong K, Moore CG, Piva SR (2011) Physical activity measured by the SenseWear Armband in women with rheumatoid arthritis. Phys Ther 91: 1367-1376.
37. Scheers T, Philippaerts R, Lefevre J (2011) Variability in physical activity patterns as measured by the SenseWear Armband: how many days are needed? Eur J Appl Physiol.
38. Clifford DB, Ances B, Costello C, Rosen-Schmidt S, Andersson M, et al. (2011) Rituximab-associated progressive multifocal leukoencephalopathy in rheumatoid arthritis. Arch Neurol 68: 1156-1164.
39. Sorensen KE, Celermajer DS, Spiegelhalter DJ, Georgakopoulos D, Robinson J, et al. (1995) Non-invasive measurement of human endothelium dependent arterial responses: accuracy and reproducibility. Br Heart J 74: 247-253.
40. Corretti MC, Anderson TJ, Benjamin EJ, Celermajer D, Charbonneau F, et al. (2002) Guidelines for the ultrasound assessment of endothelial-dependent flow-mediated vasodilation of the brachial artery: a report of the International Brachial Artery Reactivity Task Force. J Am Coll Cardiol 39: 257-265.
41. Roustit M, Cracowski JL (2012) Non-invasive assessment of skin microvascular function in humans: an insight into methods. Microcirculation 19: 47-64.
42. Snell CR, Stevens SR, Davenport TE, Van Ness JM (2013) Discriminative Validity of Metabolic and Workload Measurements to Identify Individuals With Chronic Fatigue Syndrome. Phys Ther.

43. Wasserman K, Hansen JE, Sue DY, Stringer WW, Whipp BJ (2004) Principles of exercise testing and interpretation: including pathophysiology and clinical applications. 2nd ed. Philadelphia, PA, USA: Lippincott Williams Wilkins.
44. Solberg G, Robstad B, Skjonsberg OH, Borchsenius F (2005) Respiratory gas exchange indices for estimating the anaerobic threshold. J Sports Sci Med 4: 29-36.
45. Beaver WL, Wasserman K, Whipp BJ (1986) A new method for detecting anaerobic threshold by gas exchange. J Appl Physiol (1985) 60: 2020-2027.
46. Light AR, Bateman L, Jo D, Hughen RW, Vanhaitsma TA, et al. (2011) Gene expression alterations at baseline and following moderate exercise in patients with Chronic Fatigue Syndrome and Fibromyalgia Syndrome. J Intern Med.
47. Morch K, Hanevik K, Rivenes AC, Bodtker JE, Naess H, et al. (2013) Chronic fatigue syndrome 5 years after giardiasis: differential diagnoses, characteristics and natural course. BMC Gastroenterol 13: 28.
48. Hanevik K, Kristoffersen EK, Sornes S, Morch K, Naess H, et al. (2012) Immunophenotyping in post-giardiasis functional gastrointestinal disease and chronic fatigue syndrome. BMC Infect Dis 12: 258.
