# Supplementary material for: Endothelial dysfunction in ME/CFS patients
Source: PLoS One. 2023 Feb 2;18(2):e0280942. doi: 10.1371/journal.pone.0280942 (PMC9894436; doi:10.1371/journal.pone.0280942)
Supplement: S2 File — (PDF) [file pone.0280942.s002.pdf]

Supporting file

SPSS Analysis code for General Linear Model (GLM) repeated measures, for Flow-mediated Dilation (FMD) and for Post-Occlusive Reactive Hyperemia (PORH).

```
GLM FMD_0m FMD_18m BY treatment_group
  /WSFACTOR=time 2 Simple(1)
  /MEASURE=FMD
  /CONTRAST(treatment_group)=Simple(1)
  /METHOD=SSTYPE(3)
  /PLOT=PROFILE(time*treatment_group) TYPE=LINE ERRORBAR=CI MEANREFERENCE=NO YAXIS
=AUTO
  /EMMEANS=TABLES(OVERALL)
  /EMMEANS=TABLES(treatment_group) COMPARE ADJ(LSD)
  /EMMEANS=TABLES(time) COMPARE ADJ(LSD)
  /EMMEANS=TABLES(treatment_group*time)
  /PRINT=DESCRIPTIVE ETASQ PARAMETER
  /CRITERIA=ALPHA(.05)
  /WSDESIGN=time
  /DESIGN=treatment_group.
```

```
GLM PORH_0m PORH_18m BY treatment_group
  /WSFACTOR=time 2 Simple(1)
  /MEASURE=PORH
  /CONTRAST(treatment_group)=Simple(1)
  /METHOD=SSTYPE(3)
  /PLOT=PROFILE(time*treatment_group) TYPE=LINE ERRORBAR=CI MEANREFERENCE=NO YAXIS
=AUTO
  /EMMEANS=TABLES(OVERALL)
  /EMMEANS=TABLES(treatment_group) COMPARE ADJ(LSD)
  /EMMEANS=TABLES(time) COMPARE ADJ(LSD)
  /EMMEANS=TABLES(treatment_group*time)
  /PRINT=DESCRIPTIVE ETASQ PARAMETER
  /CRITERIA=ALPHA(.05)
  /WSDESIGN=time
  /DESIGN=treatment_group.
```

This GLM analysis code was used to estimate the P-value for the interaction time*treatment group (rituximab versus placebo), for the difference between treatment groups for course of the outcome measures (FMD or PORH) from baseline to the specific time point at 18 months, taken from the table Tests for Within-subjects Effects (Greenhouse-Geisser corrected).

The effect size was calculated from the parameter estimates for 18 months, as differences between treatment groups with 95%CI.
