# Supplementary material for: Endothelial dysfunction in ME/CFS patients
Source: PLoS One. 2023 Feb 2;18(2):e0280942. doi: 10.1371/journal.pone.0280942 (PMC9894436; doi:10.1371/journal.pone.0280942)
Supplement: Attachment — Submitted filename: Response to reviewers.docx [file pone.0280942.s005.docx]

**Response to reviewers regarding the article "Endothelial dysfunction in ME/CFS patients"**

**(PONE-D-22-19007)**

We want to thank the reviewer and the editors for the positive evaluation of our manuscript, and for their valuable comments. We will respond to the comments made by the reviewer and the additional comments by the editors point by point, as requested (all references to manuscript text refer to the version with marked changes):

**Reviewer #1:**

SPECIFIC ISSUES.

1. *Lines 136-138 say that the severity of ME/CFS has been categorized according to self-reported function level and the physicians’ evaluation at the time of inclusion. However, nothing is said about how these different sources of information were combined. The authors should provide a detailed description of the protocol used to define the severity classes.*

Answer: We agree that our description of the severity categories are insufficient. We have added this text: (lines 142-145): *“The categorization into different severity groups was based on thorough clinical assessment by the physician at the time of inclusion, and supported by patient-reported function level and questionnaires.»*

The following list was used as a guideline for the clinicians (originally in Norwegian, translated here):

- *Severe ME/CFS often has a 5-10% function level.*
- *Moderate/severe ME/CFS often has an 8-10-13% function level.*
- *Moderate ME/CFS often has a 12-15-18% function level.*
- *Mild/moderate ME/CFS often has an 18-25% function level.*
- *Mild ME/CFS often has a 25-40% function level.*
- *Very severe ME/CFS is typically < 5%, and should not be included in the RituxME study*

We also submit the trial protocol as supplementary material (S1 File).

2. *Lines 202-204 say that some variables were not normally distributed, without specifying which variables. This creates some confusion. For example, some variables are first analyzed by ANCOVA methods (Table 2), which require normality, and then examined by the Wilcoxon’s test (Table 3), as they were non-normal. The authors should specify which variables are normally distributed and the test used to check normality.*

Answer: We agree with the reviewer that the description of our choice of analyses were unclear. All variables have been tested for normality using the Shapiro-Wilk´s test. For the vascular variables, FMD was normally distributed in both patients and controls, while PORH was normally distributed in patients, but not in the control group. The patient variables Fatigue Severity Scale, self-reported function level, SF-36 Physical Function and Steps per 24 h were not normally distributed.

We have specified and explained our choices of statistical tests more thoroughly in the "Statistical analyses" section (lines 207-223 and 229-233) and in the tables, and hope this is satisfactory. We have, also, reanalyzed the correlation analyses including PORH measurements with non-parametric tests, and made corresponding changes in the "Results" section (lines 295, 297 and 309-315).

The figures have been adjusted with p-values from the appropriate analyses, and with error bars corresponding to the type of analyses (mean, SD for normally distributed data, and median, IQR for data not normally distributed).

3. *Lines 205-208 say that general linear models (GLM) for repeated measures were used to assess differences in changes of the outcomes measures (FMD or PORH), from baseline to 18 months, between the randomization groups (rituximab and placebo). Although this approach is in principle correct, I was not able to understand the structure of the model, because the outcome of this analysis is displayed only by a battery of pictures. I'd welcome a traditional table with the estimates (and p-values) of the coefficients of the model, including an estimate of the heterogeneity parameter (the variance of the random effects).*

Answer: We have included the analysis syntax for the GLM repeated measures as a supporting file (S2 File), which should give the details of the model. We also added in the Figure 3 the differences with confidence intervals between groups, for FMD og PORH.

**Additional requirements by the editors:**

1. *Please ensure that your manuscript meets PLOS ONE's style requirements, including those for file naming. The PLOS ONE style templates can be found at* 
[*https://journals.plos.org/plosone/s/file?id=wjVg/PLOSOne_formatting_sample_main_body.pdf*](https://journals.plos.org/plosone/s/file?id=wjVg/PLOSOne_formatting_sample_main_body.pdf) *and* 
[*https://journals.plos.org/plosone/s/file?id=ba62/PLOSOne_formatting_sample_title_authors_affiliations.pdf*](https://journals.plos.org/plosone/s/file?id=ba62/PLOSOne_formatting_sample_title_authors_affiliations.pdf)*.*

Answer: We have made changes in the manuscript to meet PLOS ONE´s style requirements. These changes are not marked in the manuscript with marked changes.

2. *Please provide additional details regarding participant consent. In the ethics statement in the Methods and online submission information, please ensure that you have specified (1) whether consent was informed and (2) what type you obtained (for instance, written or verbal, and if verbal, how it was documented and witnessed). If your study included minors, state whether you obtained consent from parents or guardians. If the need for consent was waived by the ethics committee, please include this information.*

*If you are reporting a retrospective study of medical records or archived samples, please ensure that you have discussed whether all data were fully anonymized before you accessed them and/or whether the IRB or ethics committee waived the requirement for informed consent. If patients provided informed written consent to have data from their medical records used in research, please include this information.*

Answer: We have added a section on participant consent in the Methods section (lines 127-132).

3. *We note that the grant information you provided in the ‘Funding Information’ and ‘Financial Disclosure’ sections do not match.*

*When you resubmit, please ensure that you provide the correct grant numbers for the awards you received for your study in the ‘Funding Information’ section.*

Answer: We thank the editors for pointing this out – the discrepancies may be due to different translations of funding organizations. We have corrected the information in the "Funding information" section at resubmission.

4. *We note that you have indicated that data from this study are available upon request. PLOS only allows data to be available upon request if there are legal or ethical restrictions on sharing data publicly. For information on unacceptable data access restrictions, please see* [*http://journals.plos.org/plosone/s/data-availability#loc-unacceptable-data-access-restrictions*](http://journals.plos.org/plosone/s/data-availability#loc-unacceptable-data-access-restrictions)*.*

*In your revised cover letter, please address the following prompts:*

*a) If there are ethical or legal restrictions on sharing a de-identified data set, please explain them in detail (e.g., data contain potentially identifying or sensitive patient information) and who has imposed them (e.g., an ethics committee). Please also provide contact information for a data access committee, ethics committee, or other institutional body to which data requests may be sent.*

*b) If there are no restrictions, please upload the minimal anonymized data set necessary to replicate your study findings as either Supporting Information files or to a stable, public repository and provide us with the relevant URLs, DOIs, or accession numbers. Please see* [*http://www.bmj.com/content/340/bmj.c181.long*](http://www.bmj.com/content/340/bmj.c181.long) *for guidelines on how to de-identify and prepare clinical data for publication. For a list of acceptable repositories, please see* [*http://journals.plos.org/plosone/s/data-availability#loc-recommended-repositories*](http://journals.plos.org/plosone/s/data-availability#loc-recommended-repositories)*.*

*We will update your Data Availability statement on your behalf to reflect the information you provide.*

Answer: After re-evaluation, we find that it is acceptable to make an anonymized datafile available, and have submitted this as supplementary information (S3 File for patients and PORH controls, S4 File for patients and FMD controls).

5. *We note that you have stated that you will provide repository information for your data at acceptance. Should your manuscript be accepted for publication, we will hold it until you provide the relevant accession numbers or DOIs necessary to access your data. If you wish to make changes to your Data Availability statement, please describe these changes in your cover letter and we will update your Data Availability statement to reflect the information you provide.*

Answer: We refer to our answer above regarding data availability.

6. *We note that you have included the phrase “data not published” in your manuscript. Unfortunately, this does not meet our data sharing requirements. PLOS does not permit references to inaccessible data. We require that authors provide all relevant data within the paper, Supporting Information files, or in an acceptable, public repository. Please add a citation to support this phrase or upload the data that corresponds with these findings to a stable repository (such as Figshare or Dryad) and provide and URLs, DOIs, or accession numbers that may be used to access these data. Or, if the data are not a core part of the research being presented in your study, we ask that you remove the phrase that refers to these data.*

Answer: We have deleted two references to unpublished data/observations in lines 82-85 and lines 438-440. We have kept a brief description of exploratory FMD measures from 2014 ( lines 85-91) because these findings were relevant in the planning of the substudy described in this paper. We do not have the participants´ consent to make these raw data available in a supplementary file. If the editors find this unsatisfactory, we suggest to delete all mention of these exploratory measures (lines 85-91).

7. *Please include a separate caption for each figure in your manuscript.*

Answer: We have included a caption for each figure as requested.

In addition to the changes made upon request, we suggest to delete correlations between vascular measures and disease severity at 18 months, lines 316-319. We also made some minor changes in wording and grammar throughout the text, those changes are marked in the manuscript with marked changes, but not referenced in detail here.
